# The dark side of metal exsolution: a combined *in situ* surface spectroscopic and electrochemical study on perovskite-type cathodes for high-temperature CO_2_ electrolysis†

**DOI:** 10.1039/d5ey00013k

**Published:** 2025-03-11

**Authors:** Christian Melcher, Andreas Nenning, Florian Schrenk, Kirsten Rath, Christoph Rameshan, Alexander Karl Opitz

**Affiliations:** a TU Wien, Institute of Chemical Technologies and Analytics, Getreidemarkt 9/164-EC 1060 Vienna Austria chrisitan.melcher@tuwien.ac.at; b Montanuniversität Leoben, Chair of Physical Chemistry Franz-Josef-Strasse 18 8700 Leoben Austria

## Abstract

In solid oxide CO_2_ electrolysis cells, moderate activity and coking of the cathode are major issues that hinder commercialization of this important technology. It has been already shown that cathodes based on a mixed conducting oxide decorated with well-dispersed metal nanoparticles, which were grown *via* an exsolution process, are highly resilient to carbon deposition. Using perovskite-type oxides that contain reducible transition metals, such nanoparticles can be obtained *in situ* under sufficiently reducing conditions. However, the direct catalytic effect of exsolved metal nanoparticles on the CO_2_ splitting reaction has not yet been explored thoroughly (*e.g.* by employing well-defined model systems), thus, an in-depth understanding is still lacking. In this study, we aim at providing a crucial piece of insight into high-temperature electrochemical CO_2_ splitting on exsolution-decorated electrodes: we present the results of combined Near Ambient Pressure X-ray Photoelectron Spectroscopy (NAP-XPS) and electrochemical measurements on three different ferrite perovskites, which were employed as thin film model electrodes. The investigated materials are: La_0.6_Ca_0.4_FeO_3−*δ*_ (LCF), Nd_0.6_Ca_0.4_FeO_3−*δ*_ (NCF), and Pr_0.6_Ca_0.4_FeO_3−*δ*_ (PCF). The results obtained allow us to directly link the electrode's CO_2_ splitting activity to their surface chemistry. Especially, the electro-catalytic activity of the materials decorated with and without metallic iron nanoparticles was in focus. Our experiments reveal that in contrast to their beneficial role in H_2_O electrolysis, exsolved Fe^0^ metal particles deteriorate CO_2_ electrolysis activity. This behavior contrasts with expectations derived from earlier reports on porous samples, and is likely a consequence of the differences between the CO_2_ splitting and H_2_O splitting mechanism.

## Abstract 2

Our research reveals the deactivating effect of metal exsolution on high-temperature CO_2_ electrolysis.

## Graphical abstract

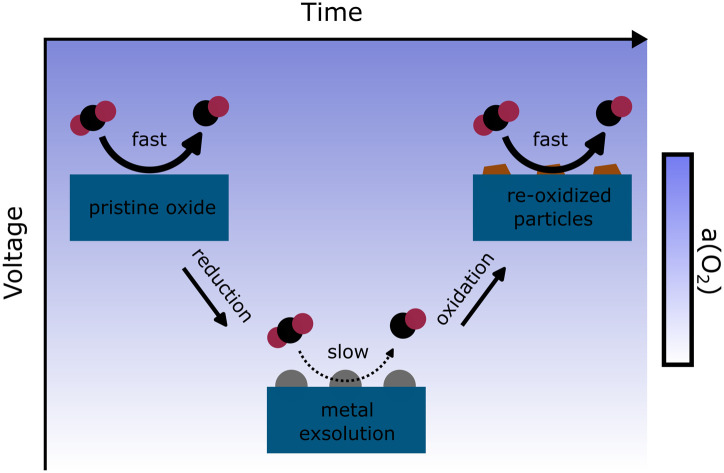

**Broader context**

The interplay between catalysis, materials science, and energy conversion is critical in addressing pressing global challenges such as climate change and carbon neutrality. High-temperature CO_2_ electrolysis is a promising approach to closing the carbon loop by converting CO_2_ into valuable chemical feedstocks or fuels. However, the role of surface chemistry and structural evolution in determining the performance and stability of catalytic materials remains poorly understood. In our work, we uncover the “dark side” of metal exsolution, which is known for enhancing catalytic activity for some reactions, by demonstrating its deactivating effects on CO_2_ splitting performance on perovskite-type oxides. Using well defined model-electrode systems, we show that CO_2_ splitting is fundamentally different from H_2_O splitting, as it relies on surface oxide reactions rather than metal-catalyzed recombination. Our findings emphasize the importance of tailored material design, particularly in controlling surface defect chemistry and lattice composition, to optimize catalytic performance for CO_2_ electrolysis. This study not only challenges conventional views on metal exsolution but also provides insights for developing advanced, coking-resistant materials for CO_2_ utilization. By addressing the mechanistic distinctions of CO_2_ and H_2_O splitting, we contribute to the development of efficient catalytic systems for sustainable energy applications.

## Introduction

With the rising demand for energy and concerns over greenhouse gas emissions, carbon neutral energy storage systems combined with renewable energy sources are crucial. Carbon conversion and utilization (CCU) present an innovative solution, utilizing renewable energy to transform CO_2_ into chemical energy carriers like syngas or methane. One notable candidate for CO_2_ conversion is direct electrochemical CO_2_ splitting *via* solid oxide electrolysis cells (SOECs) because they allow for operating temperatures up to about 900 °C, which is beneficial for breaking the bonds of this rather stable molecule and makes graphite formation thermodynamically less favorable. The faradaic efficiency for these cells is nearly 100% and the energy efficiency increases with higher temperatures.^1–3^

Well-established cathode materials for SOECs include Ni-YSZ cermets.^1,4,5^ However, these cathodes suffer from long-term degradation issues, including impurity poisoning, detrimental microstructure evolution,^6,7^ and a relatively high susceptibility towards carbon deposition, commonly referred to as coking.^8,9^ An alternative approach relies on using Ni-GDC (GDC = Gd_2_O_3_ doped CeO_2_) or pure oxides as cathode materials. In both cases, the tendency towards coking can be decreased drastically.^10,11^ However, especially for the latter, it is crucial that the material functions as a Mixed Ionic and Electronic Conductor (MIEC), since both the oxide ions as well as the electrons must be transported from/to the reaction site of CO_2_ reduction at the surface of the cathode material. This can be straightforwardly recognized by the respective cathodic half-cell reaction (which is given in eqn (1) in Kröger–Vink notation).1
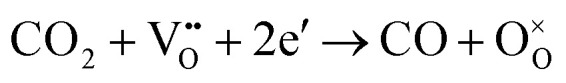
Perovskite-type oxides are a large class of mixed conducting materials that have been intensively investigated as cathodes for solid oxide electrolysis cells (SOECs).^12–17^ Recent attention to perovskite-based electrode materials arises from their promising catalytic properties and broad compositional adaptability.^18–21^ Moreover, perovskites that contain reducible transition metals such as Fe, Ni, Pd, Ru, *etc.* allow the formation of catalytically active, oxide-supported metal nanoparticles through a process commonly called exsolution.^22–27^ Exsolution catalysts have gained significant attention in heterogeneous catalysis in recent years.^20,28–31^ Firstly, the exsolved nanoparticles exhibit a lower tendency for coking, due to an increased presence of the triple phase boundary.^9^ Secondly, they are firmly anchored in the host lattice, preventing them from agglomerating.^23,32,33^

Numerous studies have demonstrated that metallic phases on the surface of an oxide, such as exsolved nanoparticles, indeed enhance the rate of certain reactions. Examples include the reverse water–gas shift reaction (rWGS),^34^ methane dry reforming (MDR),^20^ or electrochemical water splitting.^31,35,36^ Crucial for all these reactions is to obtain an improved mechanistic understanding for the enhancement of reaction rates by exsolution particles, which in these cases always involves either enhanced adsorption or recombination rates of hydrogen species on the metallic particle surfaces. For instance, the proposed mechanism for water splitting and the reason for the beneficial effect of metallic particles on the reaction rate involves a spillover mechanism.^36^ The adsorbed hydrogen on the host oxide has a high surface mobility and can easily diffuse towards the metallic surfaces where recombination into H_2_ molecules is more favored than on an oxide surface.

In the case of CO_2_ electrolysis, however, it is highly questionable whether carbonate reaction intermediates offer fast enough surface diffusion rates to enable a similar spillover mechanism. Additionally, CO_2_ splitting does not require recombination of intermediates to produce CO. Moreover, there is a general lack of well-defined model studies on the effect of exsolution on electrochemical CO_2_ splitting. Studies claiming that exsolutions enhance CO_2_ splitting kinetics were typically performed on porous electrodes, on which also variations of morphology, ionic conductivity or oxide surface composition may be responsible for the observed performance differences. On the contrary, an *in situ* NAP-XPS study on well-defined model samples for direct CO_2_ electrolysis^11^ found no significant effect of metallic nanoparticles exsolved from perovskite-type ferrites and chromites on the CO_2_ splitting rate. Thus, the beneficial effect of metal exsolution for CO_2_ splitting remains questionable and is thus the main motivation behind this study.

From the viewpoint of solid state electrochemistry, the material La_0.6_Sr_0.4_FeO_3−*δ*_ (LSF) serves as an ideal model electrode for exsolution studies, as its defect chemistry has been extensively studied and is thus well understood.^31,37,38^ Moreover, metallic iron particles can be exsolved from LSF by applying sufficiently strong cathodic bias.^35,36,39^ In the present work, we explore similar materials using the more abundant Ca as an A-site dopant instead of Sr, analyzing their electro-catalytic performance for direct electrochemical CO_2_ splitting. The materials under investigation are La_0.6_Ca_0.4_FeO_3−*δ*_ (LCF), Nd_0.6_Ca_0.4_FeO_3−*δ*_ (NCF), and Pr_0.6_Ca_0.4_FeO_3−*δ*_ (PCF). The reason for substituting La with Nd and Pr (LCF → NCF, PCF) lies in the potential valence transitions (Pr^3+^ ⇌ Pr^4+^, Nd^3+^ ⇌ Nd^2+^) and the differences in ionic radii. Especially the latter might lead to differences in exsolution behavior and stability upon reduction.

As we operate under reducing conditions (*p*O_2_ ≈ 10^−21^ bar) the oxygen vacancy concentration is primarily determined by the extrinsic Ca dopant concentration. Corresponding Brouwer diagrams exist for very similar materials like LSF.^40^ According to these, the vacancy concentration is given by 
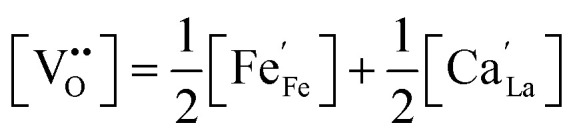
 with the same dopant concentration for all three materials (40% Ca on the A-site). Upon biasing the electrodes, we change the electron concentration in our materials in the range of 0 to 10% Fe^2+^.^40,41^ Therefore, the oxygen vacancy concentration ranges from 0.2 to 0.25 per formula unit.

The objective of this paper is therefore to investigate whether exsolved Fe-nanoparticles indeed enhance the reaction rate of high temperature CO_2_ electrolysis. To answer this question conclusively, the experimental setting was highly optimized and the experimental boundary conditions were tailored even better to this goal:

(i) Rather than using pure CO_2_ (which was needed for determination of CO_2_ conversion rates in ref. 11), we went for a CO : CO_2_ mixture of 1 : 10 to obtain a thermodynamically well-defined *p*(O_2_) for all our measurements leading to a well-defined reference point for the overpotential.

(ii) Materials wise, we focused purely on Ca-doped ferrites (*i.e.* all studied perovskite-type materials have only Fe on the B-site) with the A-site being either La, Nd or Pr (LCF, NCF or PCF). Such a focused approach is crucial to gain better mechanistic understanding of the interplay between A-site composition, Fe-exsolution and the CO_2_ splitting reaction rates. This is achieved by utilizing a lab-based Near Ambient Pressure X-Ray Spectrometer (NAP-XPS) in tandem with Electrochemical Impedance Spectroscopy (EIS) and Direct Current (DC) measurements. Through this technique, the electrochemical performance and the surface composition can be studied simultaneously.

(iii) Especially, we compare the reaction rates before and after Fe-exsolution under otherwise identical conditions (same overpotential, same *p*(O_2_), same temperature, same surface area, same sample) in one experiment.

(iv) In order to rule out as many additional contributions to the surface activity as possible, geometrically well-defined dense model electrodes were fabricated using Pulsed Laser Deposition (PLD) and photolithography. This ensures that the model electrodes have a well-defined surface area, enabling comparisons across different materials and linking electrochemical activity directly to the surface chemistry.

## Experimental methods

### Sample preparation

**Fig. 1:**
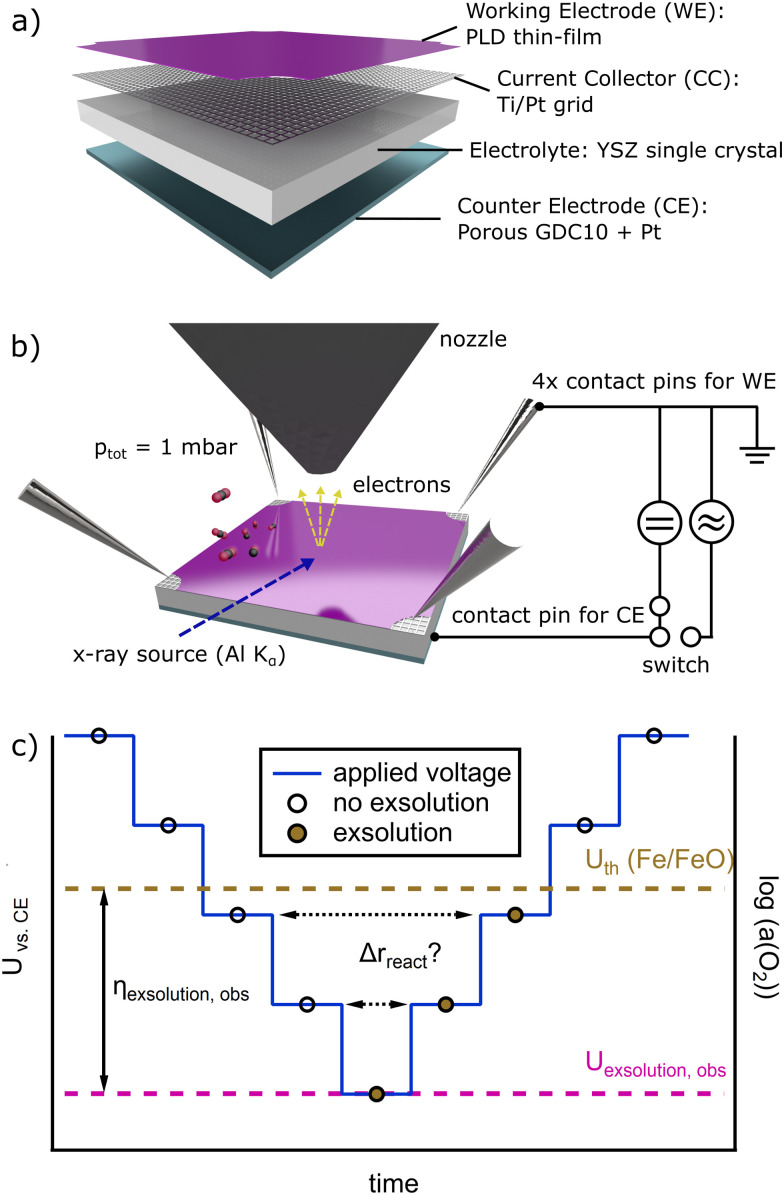
(a) Schematic drawing of the model cell. (b) Schematic drawing of the NAP-XPS/EIS sample environment. (c) Schematic of the experimental strategy for the comparison of the surface activity with and without exsolution. The potential *U vs.* CE is decreased stepwise (more negative) over time from left to right until exsolution is observed in the NAP-XPS spectra at *U*_exsolution,obs_. This voltage is typically more negative than the thermodynamic equilibrium *U*_th_(Fe/FeO) because of exsolution overpotentials (*η*_exolution,obs_ = *U*_exsolution,obs_ − *U*_th_(Fe/FeO)). When the voltage is retraced back to less cathodic values, Fe remains metallic on the surface until *U*_th_(Fe/FeO) is reached. This way, *r*_react_ at a certain set voltage can be compared with, and without exsolution.

A schematic of a typical model cell used in our NAP-XPS experiments is depicted in Fig. 1. The porous Counter Electrode (CE) was applied to the unpolished side of an (100)-oriented YSZ single crystal (9.5 mol% Y_2_O_3_ in ZrO_2_, CrysTec GmbH, Germany) by spin-coating (1000 rpm) a GDC10 paste (GDC10 = Ce_0.9_Gd_0.1_O_1.95_). The paste consisted of GDC10 powder (Treibacher Industrie AG, Austria), which was dispersed in a terpineol-based ink vehicle (FuelCellMaterials, USA) by ball milling in a 1 : 1 weight ratio. On top of the dried GDC10 paste layer, a Pt paste (Tanaka, Japan) was brushed as the electronic current collector. The CE was sintered at 1150 °C in air for 3 h to yield a porous structure. For more details on the fabrication of this type of electrode please refer to ref. 42. This type of CE was chosen since it showed exceptional surface activity.^42^

Before the actual working electrode (WE) was deposited, a 15|5 μm (mesh|strip width) Ti/Pt current collector (thickness of 5 nm Ti below 100 nm Pt) was prepared on the polished side of the YSZ single crystal electrolyte by magnetron sputtering (BalTec MED 020, Leica Microsystem GmbH, Germany) and photolithography. For a more detailed description of the photolithography process, please refer to ref. 39. Subsequently, an about 100 nm thin layer of the desired perovskite-type material (LCF, NCF or PCF) was grown by PLD atop the current collector at a *p*O_2_ of 4.0 × 10^−2^ mbar, using a KrF excimer laser (1800 pulses, 5 Hz, *λ* = 248 nm, COMPex Pro 201, Lambda Physics).^43^ This laser was employed to ablate the perovskite-type target (LCF, NCF, or PCF) that had been prepared *via* a modified Pechini method using citric acid as complexing agent,^44^ followed by isostatic pressing of the obtained powder and sintering in air at 1150 °C for 12 h. During thin film deposition, the temperature of the YSZ substrate was set to 600 °C (achieved by resistive heating and temperature control using a pyrometer).

### *In situ* NAP-XPS and electrochemical measurements

For the simultaneous investigation of surface activity and composition, electrochemical impedance spectroscopy (EIS) and direct current (DC) measurements were carried out in a NAP-XPS setup, which is sketched in Fig. 1b. In this setup, both working and counter electrodes (WE and CE) are present in one chamber without gas separation. The introduced gas consisted of a CO_2_ : CO mixture of 10 : 1. We therefore work with a thermodynamically equilibrated gas phase, which does not change in composition with higher temperatures. Note that the exact same amount of produced CO (from CO_2_ splitting at the WE) is oxidized at the CE. Therefore, the net reaction rate of the entire cell is zero and the catalytic activity cannot be derived *via* gas analysis. However, caused by our geometry (thin-film WE and porous CE) the reaction rate of CO_2_ electrolysis at the WE is rate determining. Hence, the measured current upon polarization is characteristic for the electrochemical performance of the WE. Note that for thin-films, the transport of oxide ions within the electrode material does not limit the current.^45^ Therefore, the DC current measured at a certain overpotential is directly proportional to the CO_2_ splitting reaction rate according to Faraday's law and the catalytic activity of our electrodes can be compared by evaluating the current–voltage characteristics (*I*–*V* curves).

The spectrometer utilized was a lab-based configuration equipped with a monochromatic Al-K_α_ X-ray source (μFOCUS 500 NAP, SPECS, Germany). In this setup, the hemispherical analyzer (PHOIBOS 150, SPECS, Germany) is positioned behind differentially pumped electrostatic lenses. To minimize gas phase scattering, the sample surface was positioned very close to the water-cooled nozzle, approximately 500 μm away. For a more detailed explanation of the NAP-XPS setup, please refer to ref. 46.

The sample holder, specifically designed for such experiments by Huber Scientific, consists primarily of corundum (Al_2_O_3_). The sample was fastened using four Pt pins, which were pressed against the corners of the sample through a screwing mechanism. While these pins provided electrical contact to the WE, the CE was separately connected using a Pt wire. An aperture on the backside of the sample holder permits an IR laser beam to irradiate and thus heat up the sample. The resulting temperature was monitored using a pyrometer. For an accurate temperature control, the emissivity of each individual sample was calibrated using EIS measurements as follows: the temperature dependence of the ionic conductivity of YSZ is well known.^47^ Since the geometry of the YSZ single crystal is known as well, the ohmic resistance of the sample (*R*_YSZ_ ≈ *x*-axis intercept in the Nyquist-plot) can be used to calculate the sample's temperature. This works best at relatively low temperatures (300 °C to 500 °C), since contributions of wiring and contact resistances to the total ohmic resistance can be neglected. At these lower temperatures the emissivity used by the pyrometer was adjusted to match the calculated temperature from EIS measurements. When going to higher temperatures (600 °C to 800 °C), the now calibrated pyrometer was then used for temperature control. The laser's power output was regulated through pulse width modulation according to ref. 46.

As a cleaning routine, all samples were initially heated up to 600 °C in 1 mbar O_2_ (99.999% purity, Messer, Germany) for one hour inside the NAP-XPS chamber to remove carbon contaminants before the start of the CO_2_ splitting experiment. For the subsequent NAP-XPS experiments, a total gas pressure of 1 mbar was established with a CO : CO_2_ ratio of 1 : 10 (CO_2_: 99.9995% purity, Messer, Germany; CO: 99.994% purity, Air Liquide, France) ensuring a constant and well-defined effective *p*O_2_ in the gas phase for each temperature, which determines the oxygen non-stoichiometry of our oxide materials.

Electrochemical measurements were facilitated inside the NAP-XPS chamber by feedthrough connections *via* a CF 63 flange designed for BNC connectors. For DC and EIS measurements, a Source Meter Unit (SMU, 2410 SourceMeter, Keithley Instruments, United States) and an impedance analyzer (Alpha-20 A High Performance Frequency Analyzer, Novocontrol Technologies GmbH & Co. KG, Germany) were employed, respectively. For electrochemical impedance spectroscopy (EIS), an AC voltage of 10 mV (root mean square) was applied in addition to the constant DC bias. For all experiments, the WE was connected to the mass of the XPS analyzer, and electrochemical polarization in potentiostatic mode was achieved by applying the inverse polarization to the CE. For *I*–*V* curve measurements, time-resolved potentiostatic DC measurements were conducted using the SMU.

For recording current–voltage characteristics (*I*–*V* curves), a bias voltage was applied to the model cell, which leads to cathodic polarization of the WE. This cathodic polarization was increased stepwise up to the point where Fe-exsolution was clearly visible in the Fe 2p region of the NAP-XPS spectra (roughly −275 mV, depending on the studied material). Subsequently, the cathodic polarization was decreased (*i.e.* to more positive overpotentials) to correlate the amount of metallic Fe on the surface of the electrodes to the current density. A schematic diagram of the applied voltage over the course of an experiment is shown in Fig. 1c. Time-resolved *I*_DC_ values were recorded after the bias was increased to distinguish capacitive current from faradaic current. After the *I*_DC_ values reached a steady state, EIS measurements were performed under the given bias.

As both electrodes were located in the same atmosphere, the oxygen activity in the WE *a*(O_2,WE_) could be calculated according to Nernst's equation given by eqn (2), where *η* and *U*_*vs.*CE_ correspond to the overpotential dropping at the WE and the applied voltage *U*_*vs.*CE_ with respect to the CE, respectively. *R*, *T* and *F* have their usual meanings.2
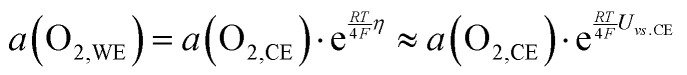
The oxygen activity of the CE *a*(O_2,CE_) is defined by the effective oxygen partial pressure of the gas phase (*p*O_2,gas_). Because the porous CE exhibits excellent surface activity compared to the WE, gas phase and CE are in equilibrium (*μ*(O_2,gas_) = *μ*(O_2,CE_)).

In principle, the overpotential *η* differs from the applied bias because the electrolyte, the CE, the wiring as well as the contact resistances contribute to the overall voltage drop (*η* = *U*_*vs.*CE_ − *I*_DC_(*R*_YSZ_ + *R*_CE_ + *R*_wire_ + *R*_contact_)). However, considering the high resistances of our thin-film electrodes, especially at the low gas pressure of 1 mbar, the former mentioned contributions can safely be neglected (*i.e. η* ≈ *U*_*vs.*CE_ holds in good approximation). The effective overpotential dropping at the WE is therefore close to the applied voltage with deviations of at most 4% (worst case at maximal applied overpotential and maximal current at 800 °C).

### Surface cation compositions

For comparing the different materials, it is important to ensure phase purity of the PLD targets. Furthermore, the materials should be similar in their surface cation composition. The former was verified using powder XRD measurements, as presented in Fig. S1 in the ESI.† The cation compositions were calculated from the XPS data using eqn (3), where *X*_*j*_, *c*_*j*_, *I*_*j*_, and *I*_BG_ represent the molar fraction, the concentration and the signal intensity of species *j*, and the intensity of the background, respectively. To enhance visualization, intensities were normalized to the total intensities of the measured cations. The terms RSF_pwdr_, TF, and *E*_kin_ correspond to experimentally determined relative sensitivity factors by Brundle *et al.*,^48^ the transmission function (which was assumed to be constant in our case), and the kinetic energy, respectively. Measured RSF_pwdr(48)_ values for La 3d, Nd 3d, Ca 2p, and Fe 2p transitions are summarized in Table S2 in the ESI.†3
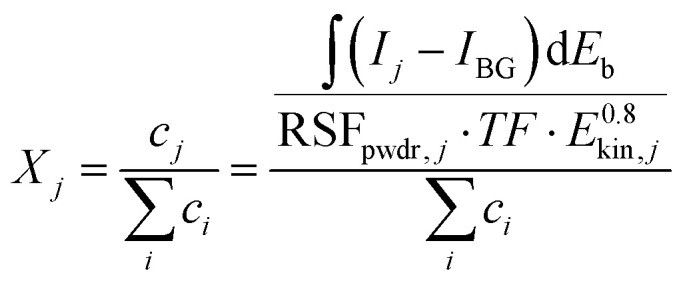


## Results

### CO_2_ electrolysis on bare electrodes without exsolutions

The following subchapters represent results of the thin-film working electrode (WE) from measurements inside the NAP-XPS chamber. Both XPS and electrochemical data were acquired simultaneously at high temperatures. Phase purity of the PLD targets was determined by analyzing powder XRD diffraction patterns (Fig. S1 in the ESI†). The patterns show the typical reflexes of orthorhombic distorted perovskites indicating successful synthesis and phase purity of the materials.^18,49–51^

#### Electrochemical characterization

**Fig. 2:**
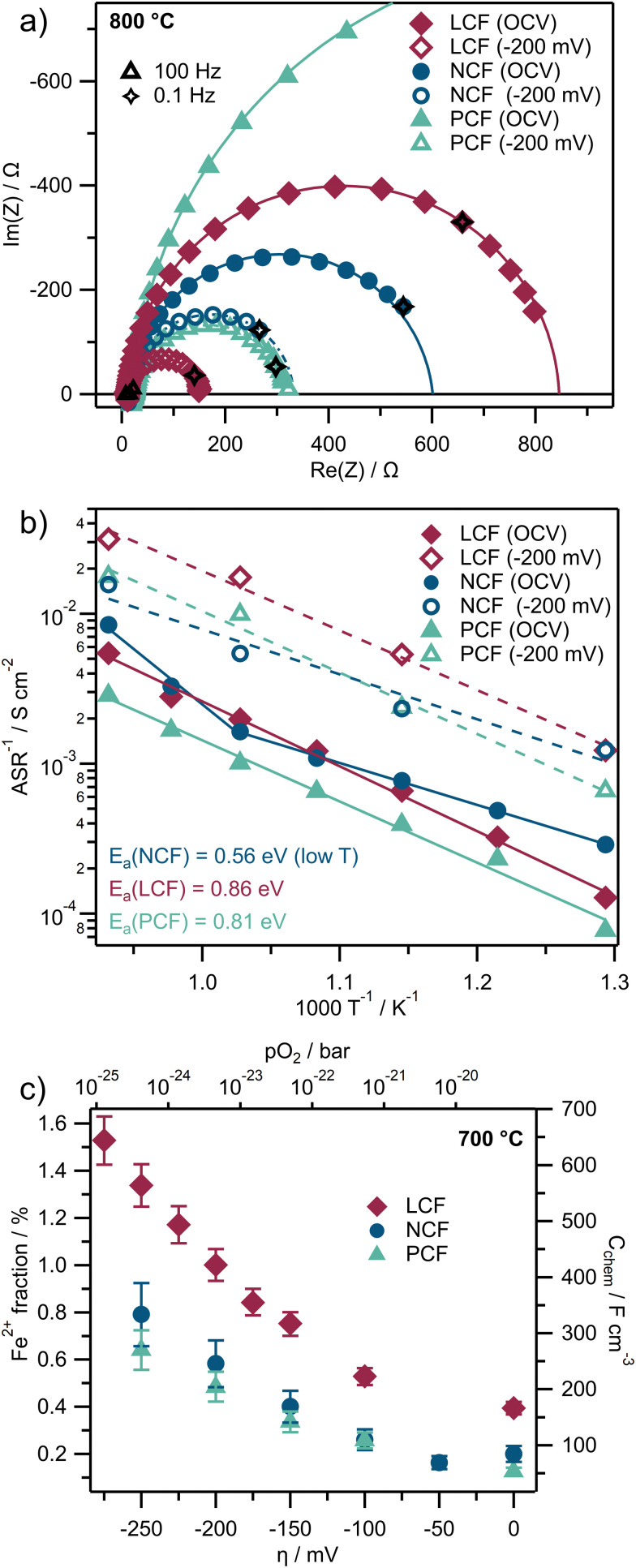
Results from electrochemical measurements taken inside the NAP-XPS chamber in a 1 mbar CO : CO_2_ atmosphere of a 1 : 10 ratio. (a) Exemplary Nyquist-plots of EIS measurements (markers) at 800 °C plus complex non-linear least squares fitting functions (lines) at OCV and under cathodic *η* of −200 mV. (b) Arrhenius-plot of inverse ASR values resulting from EIS measurements using the same legend as in (a). (c) Exemplary *C*_chem_ values and Fe^2+^ bulk fractions calculated from EIS results and at 700 °C.

Fig. 2a presents results from electrochemical impedance spectroscopy (EIS) measured at 800 °C inside the NAP-XPS chamber without normalization to the electrode geometry. Therein, solid markers indicate the values measured at OCV, while hollow markers represent values under a cathodic overpotential *η* of −200 mV *vs.* the Counter Electrode (CE) in a CO : CO_2_ atmosphere with a 1 : 10 ratio. The electrode features in the Nyquist plot show almost ideal semi-circles, which exhibit polarization resistances between 150 Ω and 1800 Ω, depending on the material and overpotential, and capacitances in the order of 10^−4^–10^−3^ F.

For the evaluation of the area specific resistance (ASR) and the chemical capacitance (*C*_chem_) values, a complex non-linear least squares fitting function is calculated using a *R*_YSZ_–*R*_ode_‖CPE equivalent circuit, which allows extracting the electrode polarization resistance *R*_ode_ (the diameter of the semicircle in the Nyquist-plot) and the capacitance *C* from the low frequency feature. For the ASR, *R*_ode_ is normalized to the geometric surface area (roughly 0.2 cm^2^).

The temperature dependence of the electrode activity is displayed in an Arrhenius plot (Fig. 2b) where the reciprocal values of the area specific resistances of *R*_ode_ (ASR^−1^) are plotted against 1000/*T* (again solid markers indicate measurements at OCV, hollow markers represent values under cathodic *η* of −200 mV *vs.* CE). The activation energies *E*_a_, which can be calculated from the slopes of linear fits of the data points in Fig. 2b (solid lines), range between 0.56 ± 0.03 eV and 0.81 ± 0.05 eV (uncertainties represent two times the standard deviation derived from the uncertainties in the slopes of the least-squares fit in the Arrhenius plot, corresponding roughly to a 95% confidence interval). These values remain largely unaffected when a cathodic overpotential is applied, however, the ASR significantly decreases under bias causing a parallel shift in the Arrhenius plots. This behavior is expected and aligns with the understanding that perovskite-type mixed conducting electrodes in reducing atmospheres typically exhibit non-linear *I*–*V* curves, which was already observed for LSF electrodes in ref. 11 and 37.

The capacitance associated with the low-frequency feature of the impedance spectra, captured in the fit as a constant phase element (CPE), represents the largest capacitance in the system. Based on our defect chemical understanding of closely related perovskites such as LSF^38^ it can be safely assumed that this capacitance corresponds to the chemical capacitance *C*_chem_ of the perovskite-type working electrodes rather than to other typically electrostatic capacitive contributions (*e.g.* originating from interfaces).

As *C*_chem_ depends on the minority charge carrier concentration^38^ and thus scales with the film thickness, the capacitances (in the order of 10^−4^–10^−3^ F) are normalized to the volume of the film (roughly 2 × 10^−6^ cm^2^), which leads to values in the order of 10^2^–10^3^ F cm^−3^ at 700 °C (see Fig. 2c). The film thickness was determined using SEM cross sections (see Fig. S11–S13 in the ESI†) and vary between 240 and 360 nm. Error bars in Fig. 2c depict the uncertainties of the *C*_chem_ values which are propagated by two times the standard deviation from SEM cross section thicknesses (roughly 10%). Uncertainties coming from impedance fitting are neglected, as they are found to be much smaller than the scatter in film thicknesses (roughly 0.2%).

As an *R*_YSZ_–*R*_ode_‖CPE circuit was used for impedance fitting, with the CPE element covering the slight deviations from an ideal semicircle,^52^ a real capacitance can be calculated in good approximation from the CPE-element fit parameters and the electrode resistance according to ref. 53. The obtained *C*_chem_ is directly proportional to the minority charge carrier concentration of the WE bulk material.^54,55^ Furthermore, the defect chemistry of the given ferrite-based perovskites in reducing atmospheres is governed by a high number of oxygen vacancies, and a comparatively small concentration of electronic point defects, which are expected to be localized to the B-site cations of the perovskite, thus appearing as Fe^2+^ ions.^41^ Under these conditions, and assuming that defect interactions play a minor role, the Fe^2+^ bulk concentration [Fe^2+^] can be calculated according to eqn (4).^38^ Here, *V*_m_ and *V*_film_ represent the molar volume and the volume of the PLD layer, respectively, while *R*, *T* and *F* retain their usual meanings.4
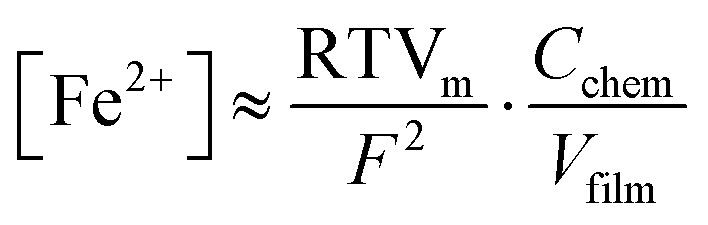
In Fig. 2c the resulting electronic minority charge carrier concentration (exemplary for 700 °C) is plotted *versus* the electrode overpotential, showing that the values for [Fe^2+^] range from roughly 0.2% to 1.6% relative to the B-site. Moreover, for all three materials [Fe^2+^] increases with cathodic overpotential. For a more comprehensive view of the ASR and *C*_chem_, heatmaps depicting them as a function of overpotential and temperature for all three materials are provided in Fig. S3 in the ESI.†

#### Surface characterization by XPS

**Fig. 3:**
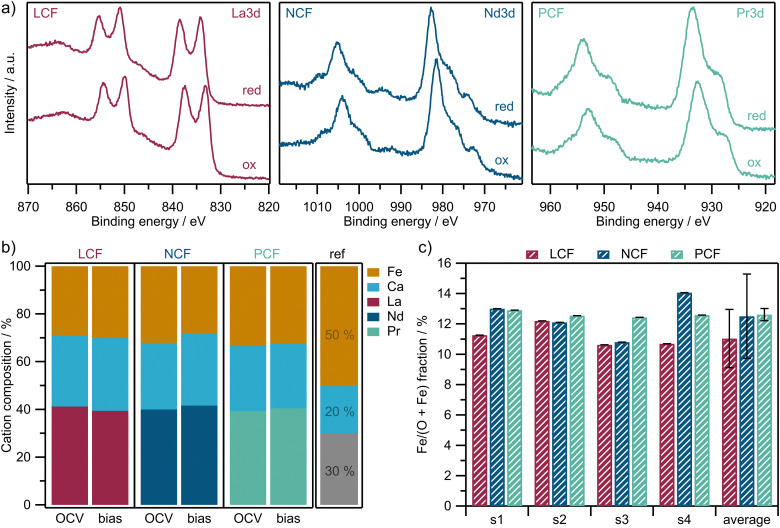
(a) Exemplary NAP-XPS spectra of the A-site cations under oxidizing (1 mbar O_2_, OCV) and reducing (1 mbar CO : CO_2_ = 1 : 10, −200 mV) conditions. (b) Normalized surface cation composition at 700 °C from *in situ* NAP-XPS measurements under OCV and −200 mV (bias). The bar labeled ‘ref’ indicates the nominal values of the bulk materials as a guide to the eye. (c) RSF and *E*_kin_ corrected Fe/(O + Fe) fractions of different samples (s1, s2, s3, s4) under oxidizing conditions at 600 °C. The error bars indicate two times the standard deviation between the four different samples of the same material.

To be able to follow the surface chemical changes, which accompany the electrochemical polarization of the WE, NAP-XPS measurements were performed. To show that the different perovskites used in this study are comparable regarding their surface composition, in the first step this spectroscopic characterization was conducted under conditions where no metallic Fe had yet been exsolved. Fig. 3a shows NAP-XPS spectra of the A-site cations under oxidizing (1 mbar O_2_, OCV) and reducing (1 mbar CO : CO_2_ = 1 : 10, −200 mV *vs.* CE) conditions. The normalized surface cation compositions are calculated according to eqn (3) in the Experimental section and are depicted in Fig. 3b (for exemplary Fe 2p spectra please see Fig. 6a in the following section). The bar on the right labeled ‘ref’ indicates the nominal cation fraction of the synthesized bulk materials 
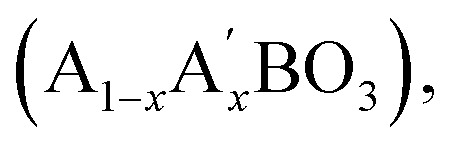
 providing a visual benchmark. The results indicate that all three materials show a lower B-site and a higher A-site cation fraction at their surface than the nominal bulk material. As an additional check, and in order to estimate reproducibility and uncertainty, the corrected Fe/(Fe + O) fractions (see eqn (3)) are calculated for different samples of the same material (denoted s1, s2, s3, s4) at 600 °C under oxidizing conditions (see Fig. 3c). Error bars (which are overlapping) indicate two times the standard deviation, which roughly corresponds to a confidence level of 95%.

In order to comprehensively assess the surface chemistry of the electrodes under *in situ* conditions, it is not only important to spectroscopically analyze the constituents of the electrode materials, but also those of potential adsorbates. From literature it is known that carbonate-type intermediates form on the electrode surface during CO_2_ electrolysis on ceria-based^9,56^ and perovskite-type electrodes.^11^ Therefore, the observation of these intermediates under conditions without Fe metal exsolution is a first important step.

**Fig. 4:**
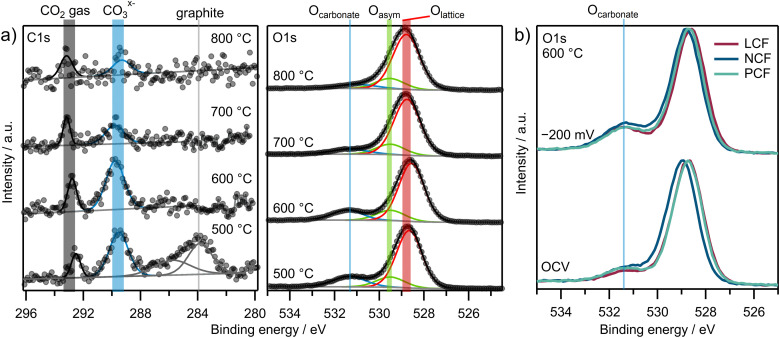
NAP-XPS spectra of (a) C 1s and O 1s regions for LCF with varying temperatures under −200 mV cathodic *η* and (b) O 1s regions of all three materials at 600 °C under OCV and −200 mV cathodic overpotential.

NAP-XPS spectra of the C 1s and O 1s regions for LCF under an overpotential of −200 mV *vs.* CE across varying temperatures are presented in Fig. 4a. For better comparability, the spectra in the C 1s region are normalized to the baseline of each individual C 1s spectrum. Three main peaks are evident: a CO_2_ gas phase peak at 292.8 eV,^11,57^ a peak related to a carbonate adsorbate at 289.5 eV,^11,57,58^ and – for the lowest temperature – an asymmetric peak at 283.8 eV corresponding to graphite-like carbon.^11,57,59–61^ The O 1s spectra in Fig. 4a display a pronounced asymmetric peak at 528.8 eV, stemming from lattice oxygen species and in part from surface oxygen species.^57,62,63^ This peak is modeled using two components: O_lattice_ and O_asym_. Additionally, a signal at 531.4 eV emerges, corresponding to an adsorbed carbonate-type species.^11,57^Fig. 4b displays the normalized intensities of the O 1s regions for all three materials at OCV and under −200 mV overpotential. The O_carbonate_ peak is evident regardless of the material and increases with cathodic overpotential.

The graphite-like carbon peak for LCF at −200 mV in the C 1s region in Fig. 4a is only visible at the lowest temperature of 500 °C. Signals from the carbonate species in both the C 1s and O 1s regions decrease above 600 °C. For further quantification of the carbonate coverage, the O 1s region is considered, as it offers a superior signal-to-noise ratio (SNR) in comparison to the C 1s spectra. However, to observe graphite-like carbon on the surface, the C 1s region is essential.

**Fig. 5:**
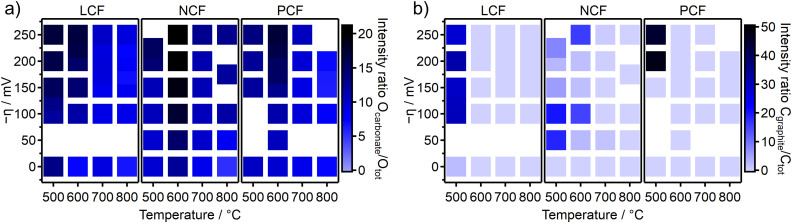
Heatmaps of NAP-XPS intensity ratios for all three materials of (a) O_carbonate_/O_tot_ and (b) C_graphite_/C_tot_. Each square represents one measurement point at a certain combination of temperature and overpotential. The color of each square indicates the intensity ratio according to the color scale on the right creating a data matrix across various temperatures and electrochemical overpotentials.

Analogous NAP-XPS spectra were acquired for all three electrode materials under various overpotentials. Fig. 5a displays heatmaps of the O_carbonate_/O_tot_ intensity ratios as a function of temperature and overpotential. Each square represents a set of temperature and overpotential and the color of each square indicates the intensity ratio. The adsorbed carbonate is observed under all conditions and the intensity ratios range from 2% to 25%. The signal intensity ratio C_graphite_/C_tot_ in Fig. 5b on the other hand is zero under most conditions and graphite-like carbon is only observed at lower temperatures (500 °C and 600 °C) and higher cathodic overpotentials.

### CO_2_ electrolysis on electrodes decorated with Fe exsolutions

#### Electrochemically triggering Fe^0^ exsolution

After studying the CO_2_ electrolysis kinetics and surface chemistry of the undecorated electrodes, we now want to explore both on the respective exsolution-decorated surfaces. For this, in a first step the electrodes must be polarized to overpotentials that are cathodic enough to trigger Fe metal particle exsolution (see Fig. 1c in Experimental section). This initial exsolution was monitored by *in situ* NAP-XPS.

**Fig. 6:**
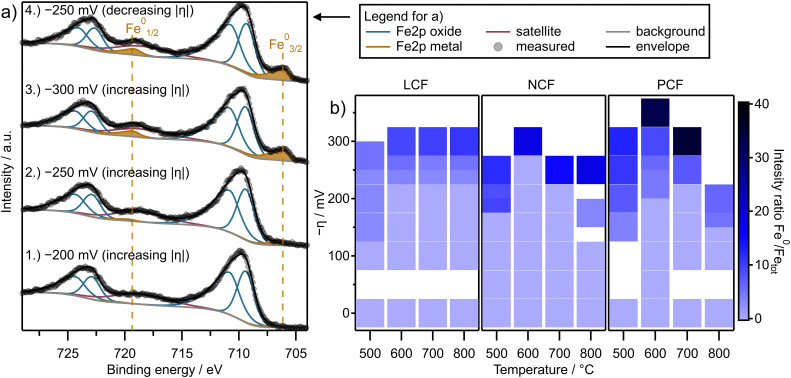
(a) Exemplary Fe 2p NAP-XPS spectra of LCF at 700 °C under varying cathodic overpotentials for increasing (spectra no. 1–3) and decreasing (spectrum no. 4) |*η*|. (b) Heatmaps for the increasing |*η*| branch showing the measured fraction of metallic iron, thus indicating the degree of Fe exsolution for the pristine films of all three materials. Each square in (b) represents one measurement point at a certain combination of temperature and overpotential. The color of each square indicates the intensity ratio according to the color scale on the right creating a data matrix across various temperatures and electrochemical overpotentials.

Fig. 6a shows the Fe 2p region of the measurements on LCF at 700 °C under varying cathodic overpotentials (*η*). Note that the XPS fitting is not sufficient to separate Fe^2+^ from Fe^3+^. Therefore, only the sum of both is depicted as Fe oxide which is modeled using two constrained peaks to match the asymmetric shape of the Fe oxide peak. The Fe metal peak on the other hand is clearly separated and well de-convoluted using XPS fitting.

Initially, *η* was set from −200 mV to −300 mV in a stepwise manner (increasing |*η*|, spectra no. 1–3 in Fig. 6a). Subsequently, *η* was retraced back to −250 mV (decreasing |*η*|, spectrum no. 4 in Fig. 6a). It is evident that initially, under overpotentials slightly less cathodic than or equal to −250 mV, no metallic iron is present on the surface, even though we would expect it to be stable under these conditions. Only under overpotentials more cathodic than −250 mV, two additional peaks appear at 707 eV and 719 eV for the Fe 2p_3/2_ and Fe 2p_1/2_ branches. Their binding energies perfectly match metallic Fe,^31,64,65^ indicating the successful exsolution of Fe^0^ particles. These additional peaks remain unaffected when *η* is retraced back from a maximum −300 mV to −250 mV (decreasing |*η*|, spectrum no. 4 in Fig. 6a) and the exsolved iron remains metallic even under conditions at which no Fe^0^ was observed during the initial reduction (compare spectra no. 2 and 4 in Fig. 6a). This demonstrates that the initial formation of exsolved particles requires an additional exsolution overpotential *η*_ex_, which is in line with our prior experience with similar materials as well as with literature reports. Consequently, the experimental strategy proposed in Fig. 1c is directly applicable to the materials studied here. This offers the opportunity that two different states of the material can be observed under otherwise identical conditions (same overpotential and temperature): one with and one without Fe exsolution. In other words, a hysteresis-like behavior emerges regarding the Fe^0^ content on the surface. As a next step, the surface activities of these different stages (with and without exsolved Fe^0^) will be compared in the following chapter by looking at the current voltage characteristics (*I*–*V* curve).

The tendency for Fe exsolution of different materials can be an important factor for the kinetics of CO_2_ splitting. Therefore, a comprehensive visual representation of the relative Fe^0^ amount is illustrated using heatmaps in Fig. 6b for the increasing |*η*| branch thus depicting the Fe exsolution tendency of the pristine materials. Here, the NAP-XPS signal intensity ratios of Fe^0^/Fe_tot_ (Fe_tot_ = Fe^0^ + Fe^II^ + Fe^III^) from the Fe 2p region are displayed as a function of temperature and overpotential for all three materials. Caused by uncertainties from XPS fitting, the Fe^0^/Fe_tot_ threshold indicating significant amounts of exsolution was found to be 3%. Therefore, Fe^0^/Fe_tot_ ratios below this 3% threshold are assumed to be insignificant, which is taken into account in the definition of the color bar in Fig. 6b. The results show that the amount of metallic Fe is strongly increased by overpotential. Temperature on the other hand seems to play only a minor role.

#### Correlation between CO_2_ splitting activity and Fe metal exsolution

**Fig. 7:**
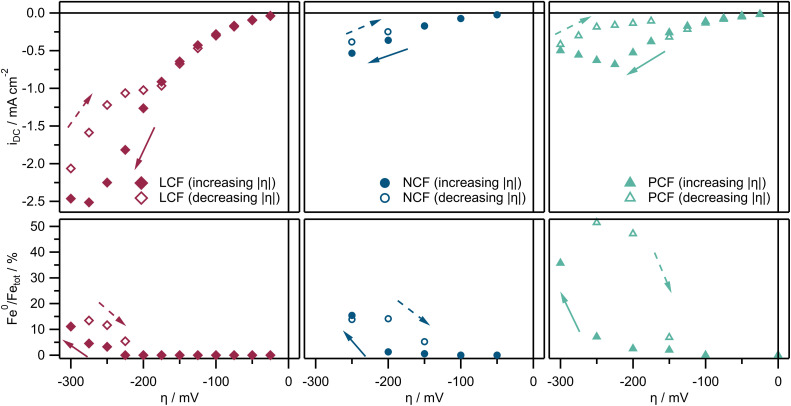
Top *y*-axis: Current–voltage characteristics (*I*–*V* curves) for all three materials at 700 °C showing the current densities *i*_DC_ as a function of *η*. Bottom *y*-axis: Corresponding Fe^0^/Fe_tot_ ratios calculated from Fe 2p NAP-XPS spectra also as a function of *η*. For all subplots, the solid markers represent values when the cathodic overpotential is ramped up initially (increasing |*η*|) while the hollow markers represent values when the cathodic overpotential is subsequently decreased (decreasing |*η*|).

Fig. 7 shows the current–voltage characteristics (*I*–*V* curves, top *y*-axis) of all three materials at 700 °C, where the current densities *i*_DC_ (*I*_DC_ normalized to the WE area) are plotted against *η*. As the current is limited by the surface exchange reaction (CO_2_ reduction), the current density directly depicts the catalytic performance of the electrodes. The solid markers show *i*_DC_ values when |*η*| is initially increased (more negative), while the hollow markers are values when |*η*| is subsequently decreased after the first reduction. In the increasing |*η*| direction up to −200 mV, all materials display a non-linear *I*–*V* curve, demonstrating an exponential behavior under moderate cathodic overpotentials. For LCF and PCF, as the cathodic overpotential is further increased, *i*_DC_ values start to differ from this exponential behavior. This deviation is observed for *η* values more negative than −275 mV and −225 mV, respectively. After that, when the cathodic polarization is subsequently decreased again (less negative), the current density exhibits a significant decline across all materials and the return curve does not follow the original *I*–*V* characteristics. However, once a moderate overpotential is reached again (−175 mV for LCF, −150 mV for NCF and PCF), the *I*–*V* behavior switches back to the initial (exponential) curve that was observed upon increasing |*η*|. As a result, a hysteresis-like feature is observed for the part of the *I*–*V* curve measured under strong cathodic polarization.

Beneath the *I*–*V* curve of each electrode in Fig. 7 the fraction of metallic Fe^0^ on the surface of the electrodes (Fe^0^/Fe_tot_ as calculated from NAP-XPS measurements) is plotted also as a function of the applied overpotential. As was already described in Fig. 6a, all materials show a hysteresis-like relationship with respect to the Fe^0^ fraction as well, and the overpotential region of the Fe^0^ fraction hysteresis loop perfectly coincides with the *I*–*V* hysteresis. Similar *I*–*V* and Fe^0^ fraction curves for all three materials across the entire measured temperature range (500 °C to 800 °C) can be found in Fig. S4 in the ESI.† The results show that the above-described hysteresis in Fe^0^ fraction and the electrode de-activation are reproducible for all materials and temperatures.

## Discussion

In the current study, three aspects are in focus, which will be discussed in the following. Firstly, we compare the electrochemical CO_2_ splitting performance of ferrite-based perovskites with different A-site cations. Secondly, we *in situ* explore the onset conditions of Fe exsolution depending on the A-site composition. And thirdly, we aim at correlating the CO_2_ splitting kinetics of different ferrite-based electrodes with the appearance of Fe^0^ exsolutions to gain further insights into the high temperature CO_2_ electrolysis kinetics and point out fundamental differences with the water splitting reaction.

### Effect of A-site composition on CO_2_ splitting performance

The investigation of the surface elemental composition shown in Fig. 3b revealed that all materials had similarly strong surface enrichment of their A-site elements, which is a well-known behavior of perovskite-type materials under such conditions.^39,66–70^ The observed variations in surface Fe content among the different materials in Fig. 3b can be regarded as small and statistically insignificant. This is demonstrated in Fig. 3c, where the Fe/(Fe + O) fractions are plotted for different samples of each material. First, the error bars in Fig. 3c (twice the standard deviation) overlap, indicating that the B-site content on the surface does not differ significantly between LCF, NCF and PCF (confidence level of roughly 95%). Second, the statistical variations in the B-site content of the PLD layers for each material in Fig. 3c are higher than the differences in B-site composition between the materials in Fig. 3b. This suggests that the A : B ratio of the surface cations is consistent across different samples when compared to the statistical variation of the films. Hence, the main surface chemical factors that were varied in this study are restricted to the elements occupying the A-site and the Fe oxidation state. This well-defined approach is essential to ensure that the specific electrode parameters being varied are precisely known, enabling robust and reliable conclusions about electrode kinetics.

The electrochemical impedance (Fig. 2 and Fig. S2, S3, ESI†) as well as DC measurements (Fig. 7 and Fig. S4, ESI†) show a superior electrochemical activity of LCF, compared to NCF and PCF, across the entire measured temperature range. This difference in electrochemical activity of the three perovskite-type electrodes, while exhibiting virtually identical A : B cation ratio at the surface, may be explained by two effects: either, the rare earth metal on the A-site also contributes redox activity, or the different size of La, Nd, and Pr affects the defect chemistry of the perovskite.

By looking at the NAP-XPS spectra of the A-site elements (La 3d, Nd 3d and Pr 3d) in Fig. 3a it becomes evident that no changes regarding the shapes of the spectra are observed upon electrochemical reduction. Only a binding energy shift of the entire A-site spectrum is observed, which can be explained by the Fermi-level freely moving through the band gap without being pinned by a redox active state.^39^ Since the shapes of the spectra are not altered at all after the reduction, a redox change can be ruled out for all three A-site elements. Furthermore, since La can be expected to be the least redox active element of the three, but LCF offers the fastest kinetics, the first option – *i.e.* the rare earth element contributing redox activity – can be excluded as an explanation for the materials’ different electrochemical activity.

Hence, the size effect of the A-site cations may be the more suitable explanation. Indeed, the observed activity trend (LCF > PCF ≈ NCF) correlates with the ionic radii of the elements: La > Pr ≈ Nd. The larger ionic radius of La^3+^ leads to a lattice expansion, as evidenced by XRD, which reveals that LCF exhibits the largest unit cell (see Fig. S1 and Table S1 in ESI†). This lattice expansion can increase the reducibility of the perovskite and enhance its surface reactivity, a phenomenon previously demonstrated for similar perovskites through ^18^O tracer exchange experiments on intentionally tensile-strained cobaltite perovskite thin films.^71^ Similar A-site size effects on the reducibility of perovskite-type ferrites were reported using thermogravimetry.^72^ Also, for the materials studied in the present case, an easier reducibility of LCF is supported by EIS measurements, since LCF indeed exhibits the largest chemical capacitance *C*_chem_. Since *C*_chem_ is directly proportional to the minority charge carrier, this indicates a larger n-type electron concentration (*i.e.* Fe^2+^ concentration)^54^ and thus a higher degree of reduction of LCF than the other two oxides under otherwise identical conditions (compare Fig. 2c and Fig. S3 in the ESI†). From the viewpoint of LCF exhibiting a larger lattice constant this behavior seems to be plausible, as a lattice with larger unit cell volume can easier accommodate electronic charge carriers that localize at the B-site cation thus appearing as Fe^2+^, which is larger than Fe^3+^. Interestingly, Fe metal exsolution (which does not necessarily correlate to Fe^3+^/Fe^2+^ reduction) on the other hand is more pronounced for NCF and PCF. This phenomenon might be attributed to different types/degrees of lattice strain caused by different sizes of the A-site cations. Caused by the relatively smaller Nd^3+^ and Pr^3+^ cations compared to La^3+^, NCF and PCF exhibit more lattice distortion (and thus more local strain) compared to LCF. Strain has already been demonstrated to be able to strongly affect the exsolution behavior of perovskite films,^73^ and hence misfit-strain induced by size-mismatch of the A-site element may be a possible explanation for the observed differences of LCF, NCF, and PCF.

### Formation and electrochemical redox-switching of Fe exsolutions

As described in the Results section, we observed a hysteresis loop in the occurrence of the Fe^0^ metal fraction at the electrode surface, depending on whether we drive the overpotential from or to more reducing conditions (see *I*–*V*-curve in Fig. 7): when the electrolysis overpotential is initially increased, the formation of metallic Fe^0^ starts at around −275 mV. Upon retracing the cathodic overpotential back to lower values, the reduced Fe^0^ remains metallic in a broader voltage range and is re-oxidized between −175 mV and −150 mV. This approach allowed us to capture two distinct *i*_DC_ values corresponding to two different surface states of the electrode under otherwise identical conditions (same overpotential and temperature): a pristine one with (almost) no Fe^0^ present on the surface, and another one with a considerable Fe^0^ surface fraction after reduction.

Regarding the effective *p*O_2_ of the initial formation of exsolution particles, the measurements are in line with literature. Very similar exsolution onset points of Fe^0^ nanoparticles were previously observed for NCF under steam electrolysis conditions in ref. 74. The Fe^0^/FeO equilibrium at 700 °C corresponds to an equivalent *p*O_2_ of 2.7 × 10^−22^ bar,^75^ which corresponds to a Nernst voltage of −114 mV *vs.* CE in a 1 : 10 CO : CO_2_ atmosphere (*p*O_2_ of 6.4 × 10^−20^ bar). The fact that we observe the Fe^0^ exsolution onset at substantially more cathodic overpotentials (about −275 mV) strongly suggests that the initial exsolution process is kinetically hampered – *e.g.* by diffusion of involved species, electron transfer of the required iron reduction reaction, particle nucleation, and particle growth – and is in line with previous reports.^74,76^

When comparing the exsolution onsets of the different materials, subtle differences emerge by looking at Fig. 6b. Firstly, LCF appears to exhibit a slightly lower tendency for Fe exsolution since the Fe^0^ fraction at high overpotentials is rather low. Secondly, while LCF seems to exsolve in a more gradual manner, both NCF and PCF display a more abrupt increase in the Fe^0^ fraction upon reaching a certain overpotential threshold at around −275 mV.

Analyzing the *i*_DC_ values of both states in Fig. 7, a clearly negative correlation between *i*_DC_ and Fe^0^ fraction emerges, since the magnitude of the current is decreasing with increasing Fe^0^ fraction. The onsets of the hysteresis loops of both the *i*_DC_ values and Fe^0^ fractions also coincide almost perfectly. Furthermore, the overpotential for the exsolution onset is within the range where the *i*_DC_ values start to deviate from an exponential behavior for LCF and PCF. Since gas diffusion limitation can be safely excluded for thin films in an atmosphere of 1 mbar,^77^ this behavior suggests the onset of a surface de-activation process. This clearly suggests that Fe exsolution appears to affect the reaction rate, but in a detrimental manner.

The negative correlation between |*i*_DC_| and the Fe^0^ fraction is reproducibly demonstrated for all three investigated materials across a wide temperature range. Analogous results at different temperatures are provided in the ESI† (Fig. S4). The consistent manifestation of this phenomenon is noteworthy: a hysteresis-like behavior for both the current density and the Fe^0^ amount is evident across almost all material and temperature combinations, along with the negative correlation between them.

Furthermore, quantification using electrochemical (bulk) data revealed that for PCF (which exhibits by far the highest Fe^0^ fraction) at 700 °C, roughly 23% of the total Fe atoms present in the lattice were reduced to metal (see Table S3 in ESI†). Given the magnitude of these B-site vacancy concentrations, it seems improbable for the perovskite lattice to remain stable.^32,41^ De-activation caused by partial decomposition of the perovskite host would indeed be a plausible explanation at first glance. However, this does not necessarily seem to be the case here. Remarkably, once the exsolved Fe^0^ is re-oxidized, the surface activity is mostly recovered. This observation appears surprising, and explanations for this phenomenon remain speculative. Owing to the complexity of the observed effect, a detailed explanation is beyond the scope of this paper. Nevertheless, some thoughts deserve attention at this point: on the one hand, (at least partial) reversible exsolution could be an explanation for the re-activation upon re-oxidation. However, from what is known from literature, this seems unlikely given that exsolution – at least at the rather moderate temperatures we applied here – is typically considered to a great extent irreversible.^32,36^ This also suggests that the decrease in activity upon exsolution is not a sheer effect of losing surface area due to coverage by the exsolved metal particles, since the oxidized particles still remain after re-oxidation. Possibly, the oxidation state of the surface-decorating particles indirectly affects the reactivity of the perovskite surface, *e.g. via* triggering work function changes of the oxide. Another possibility would be a change in morphology of the particle after oxidation caused by a sudden change in the nanoparticle/perovskite interface energy. In addition, the evolution of surface roughness upon exsolution and re-oxidation may also affect the net activity of the electrode. SEM measurements revealed pronounced morphological changes of the thin-film electrodes when comparing post-measurement samples with a pristine one (see Fig. S7–S10 in the ESI†). However, the recovery of electrode surface activity upon particle re-oxidation can only hardly be explained to the full extent from the data available so far, and additional experiments are required to draw clear conclusions.

The de-activation of the electrodes for CO_2_ splitting upon Fe^0^ exsolution seems surprising at first glance, since the beneficial effect of exsolution was demonstrated for many reactions *e.g.* steam electrolysis.^36,74^ However, the reaction mechanisms for CO_2_ and H_2_O splitting differ significantly. In H_2_O splitting, the metal particles primarily facilitate the recombination of two neutrally adsorbed hydrogen atoms H_ad_ to form H_2(36)_. In contrast, for CO_2_ splitting the presence of a metal appears not to enhance the desorption process of CO. Instead, the entire reaction seems to proceed entirely on the oxide surface. Its rate-limiting step is likely the conversion of a carbonate-type intermediate, which involves an electron transfer from the oxide to the carbonate.^11^ This carbonate intermediate was also observed in the present study and will be discussed in detail in the following section.

### Carbon surface chemistry on perovskite-type ferrites

Looking at the C 1s and O 1s regions in Fig. 4 and 5, all three materials exhibit the expected carbonate-type species, which previous works identified as the reaction intermediate of CO_2_ electrolysis on mixed conducting electrodes.^11,57^ In these studies, it is argued that CO_2_ adsorbs to an oxygen vacancy and electrons are provided by the B-site of the perovskite. The sum of all elementary steps involved in the formation of the carbonate-radical intermediate can be expressed as in eqn (5).5
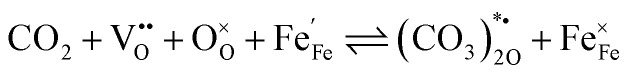
For the decomposition of this intermediate to CO and oxide ions, another electron transfer is necessary, which is regarded as rate limiting^11,57^ – see eqn (6).6
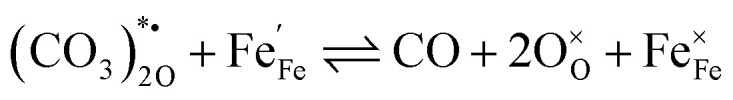
Here, we assume that the carbonate-radical intermediate is a bidentate species (
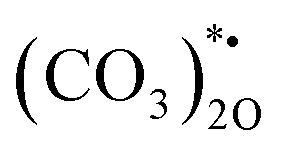
 in Kröger–Vink notation, with the asterisk denoting its radical character; using a notation of absolute charges this corresponds to (CO_3_)^3−^*). Please note that this explanation is only one possible model that is in accordance with the experimental data available so far, and that alternative binding geometries of the intermediate may also be possible. For clarification, further experimental validation would be required, which is way beyond the scope of the present work. What is important to note is that the rate of both above-described processes, the formation and conversion of the carbonate intermediate, depend on the electrode's electron concentration 
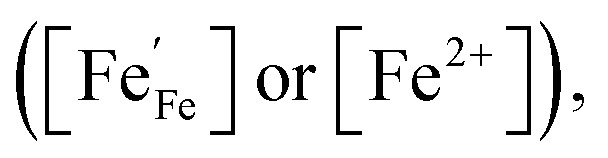
 which relates to the materials chemical capacitance (see eqn (4)^38^). Since LCF exhibits the highest chemical capacitance (see Fig. 2c), it seems plausible that also the reaction rate using LCF as the cathode is the highest among the three materials (see Fig. 2b and 7). Interestingly, no pronounced spectral features for Fe^2+^ were found in the Fe 2p spectra in Fig. 6a. Given that the bulk concentration of Fe^2+^ determined from the chemical capacitance is below 5% (Fig. 2c), its concentration is most likely below the detection limit if the surface is not much easier to reduce in terms of Fe^3+^ to Fe^2+^ reduction.

To put the NAP-XPS intensity ratios O_carbonate_/O_tot_ into perspective, the carbonate surface coverage *θ*_carbonate_ is roughly approximated by a simple calculation. With an inelastic mean free path of the O 1s photoelectrons of 2 nm^78^ and a bulk oxygen density of 5 × 10^22^ oxygen atoms cm^−3^, the bulk O 1s signal stems from approximately 10^16^ oxygen atoms cm^−2^. When further considering the C : O ratio of 1 : 3 of the carbonate, its surface concentration can be estimated by eqn (7). The maximum carbonate intensity in Fig. 5a corresponds to a coverage of about 63% relative to the unit cell density of [100] oriented film (3 × 10^15^ unit cells cm^−2^). Therefore, we can assume that already more than half of a monolayer is present at 600 °C and high overpotential. While this approximation is subject to uncertainties, it still indicates a relatively high carbonate coverage, supporting the idea that carbonate conversion is the rate-limiting step.7
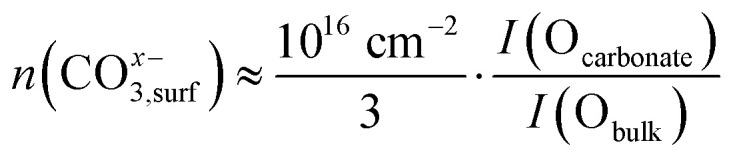
8
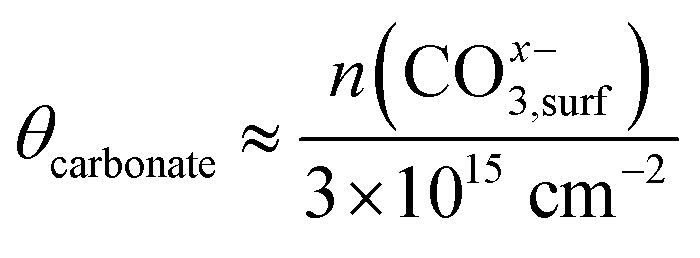
Interestingly, the carbonate coverage is not highest at the lowest studied temperature of 500 °C, where pure adsorption would typically dominate. This suggests a more complex temperature dependence with potentially also point defects contributing to the temperature dependence of the carbonate-type intermediate. For example, the lower 
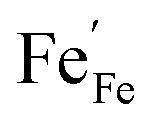
 and 
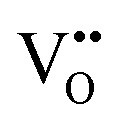
 concentrations at lower temperatures might explain the uncommon relationship between temperature and carbonate coverage.

Coking was primarily observed at 500 °C and high overpotentials (Fig. 4 and 5), which might further affect the carbonate coverage on the oxide surface since some adsorption sites might be blocked. At higher temperatures graphite-like carbon does not form, confirming the coking resistance of these materials. This is consistent with the findings of Skafte *et al.*,^9^ who observed that high oxygen vacancy concentrations and carbonate coverages on the surface delay graphite-like carbon formation.

### Effect of metal exsolution: H_2_O *vs.* CO_2_ splitting

**Fig. 8:**
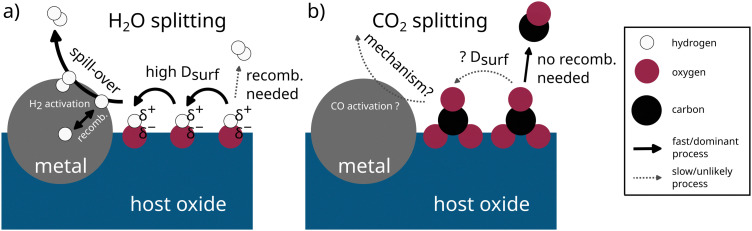
Sketch for comparing the key pathways for (a) H_2_O and (b) CO_2_ splitting. Solid black arrows depict the fast/dominant processes, dashed grey arrows depict slow/unlikely processes.

By comparing the gathered information for CO_2_ splitting with what is known for water splitting, substantial differences emerge. Fe exsolution is known to enhance the surface activity of perovskite-type oxide electrodes for H_2_O splitting.^35,36,39,74^ The reason is schematically depicted in Fig. 8a. For H_2_ to form, a recombination of two neutrally charged hydrogen species is needed. However, on the oxide surface, adsorbed hydrogen exists as positively charged OH_ads_ groups. Therefore, for recombination to occur, a simultaneous charge transfer to two adjacent OH_ads_ groups would be necessary. If a metallic surface is present nearby, an alternative pathway to this unlikely process can emerge. Because the adsorbed intermediates (OH_ads_) are likely to exhibit a large surface diffusion coefficient, they are able to move towards the metallic surface. Here, the adsorbed species H_ads_ is neutrally charged and the recombination to form H_2,ads_ is more favored.^36^

For CO_2_ splitting in Fig. 8b, the reaction proceeds differently. First, the carbonate intermediate – especially in the case of a carbonate bidentate^11^ – is probably significantly less mobile on the oxides surface and therefore might not be able to diffuse towards the exsolved nanoparticle with significant rate. Second, for CO formation, no recombination of adsorbed species is required. Therefore, the reaction on the oxide surface is probably still the dominant route for CO formation. Thus, no beneficial effect of metallic nanoparticles is observed.

## Conclusion

In this fundamental study on well-defined perovskite-type model electrodes for direct electrochemical CO_2_ splitting, we analyzed the electrochemical performance of the following materials: La_0.6_Ca_0.4_FeO_3−*δ*_ (LCF), Nd_0.6_Ca_0.4_FeO_3−*δ*_ (NCF) and Pr_0.6_Ca_0.4_FeO_3−*δ*_ (PCF). Correlations between the surface chemistry, A-site compositions, Fe exsolution and reaction rates were examined. The main takeaways from this research are:

• LCF displayed superior surface activity and the highest chemical capacitance. For all A-site elements, no changes in valence states were observed using NAP-XPS, confirming that the A-site predominantly acts as a structural provider for the materials. The key aspect for LCF's superior activity may therefore be the higher ionic radius of La^3+^ compared to Nd^3+^ and Pr^3+^. With larger A-site cations the perovskite lattice expands, thus being able to accommodate higher concentrations of n-type electronic charge carriers (*i.e.* higher concentrations of the larger Fe^2+^). Consequently, LCF exhibits more Fe^2+^, while NCF and PCF tend to exsolve higher amounts of metallic Fe^0^ under the same conditions.

• While Fe exsolution had been proven beneficial for high-temperature H_2_O splitting, it consistently exhibits a de-activating effect on direct electrochemical CO_2_ splitting in our study. This observation is validated by the tandem hysteresis behavior observed in current density and Fe^0^ fraction measured by *in situ* NAP-XPS. A plausible explanation for this substantial difference between H_2_O and CO_2_ splitting lies in the fundamental differences in their reaction mechanisms. For H_2_O splitting, the recombination of neutrally adsorbed hydrogen on the surface is accelerated strongly by metal particles *via* a spillover mechanism. In contrast, for CO formation in CO_2_ splitting, no such recombination is required and the reaction occurs on the oxide surface rather than on the metal. Additionally, the surface diffusivity of carbonate intermediates is probably quite low, preventing them from migrating towards the metallic nanoparticles.

• Using NAP-XPS, the well-known carbonate-type intermediate was detected. Notably, all three studied perovskite-type oxide electrodes showed excellent coking resistance, as graphite-like carbon was only identified in the lower temperature range of 500 °C to 600 °C. At higher temperatures, no graphite-like carbon was observed, demonstrating their potential as low-degrading electrode materials.

In conclusion, our research underscores the critical role of fundamental studies on well-defined model samples, which are essential for understanding reaction dynamics and the interplay between chemical surface states and electrochemical activity. Moreover, it is crucial to recognize that correlations observed for specific reactions may not apply universally, as demonstrated by the differing impact of Fe exsolution on H_2_O splitting *versus* direct CO_2_ splitting.

## Data availability

Data for this article, including raw impedance data, XPS spectra, cation compositions, *U–I* curves and heatmaps, are available at TU Wien Research Data at https://10.48436/vj57v-wn613.org.

## Conflicts of interest

There are no conflicts of interest to declare.

## Supplementary Material

EY-003-D5EY00013K-s001
