# Supplementary material for: The dark side of metal exsolution: a combined in situ surface spectroscopic and electrochemical study on perovskite-type cathodes for high-temperature CO2 electrolysis
Source: EES Catal. 2025 Mar 11;3(3):550–65. doi: 10.1039/d5ey00013k (PMC11894520; doi:10.1039/d5ey00013k)
Supplement: EY-003-D5EY00013K-s001 [file EY-003-D5EY00013K-s001.pdf]

Supplementary Information (SI) for EES Catalysis.
This journal is © The Royal Society of Chemistry 2025

Supporting Information

The Dark Side of Metal Exsolution: A Combined In-Situ Surface Spectroscopic and Electrochemical Study on Perovskite-Type Cathodes for High-Temperature CO_2 Electrolysis

Christian Melcher[1], Andreas Nenning[1], Florian Schrenk[2], Kirsten Rath[1], Christoph Rameshan[2], Alexander K. Opitz[1]

[1]TU Wien, Institute of Chemical Technologies and Analytics

[2]Montanuniversität Leoben, Chair of Physical Chemistry

X-ray diffraction of the PLD-targets

Figure S1 depicts powder XRD patterns of the PLD-targets. For clarity, the pseudo cubic Miller indices are depicted. Note that the minor peaks are part of the orthorhombic perovskite structure, as indicated by the markings labeled as ‘o’. All three materials show patterns of an orthorhombic perovskite structure without clearly visible impurities.

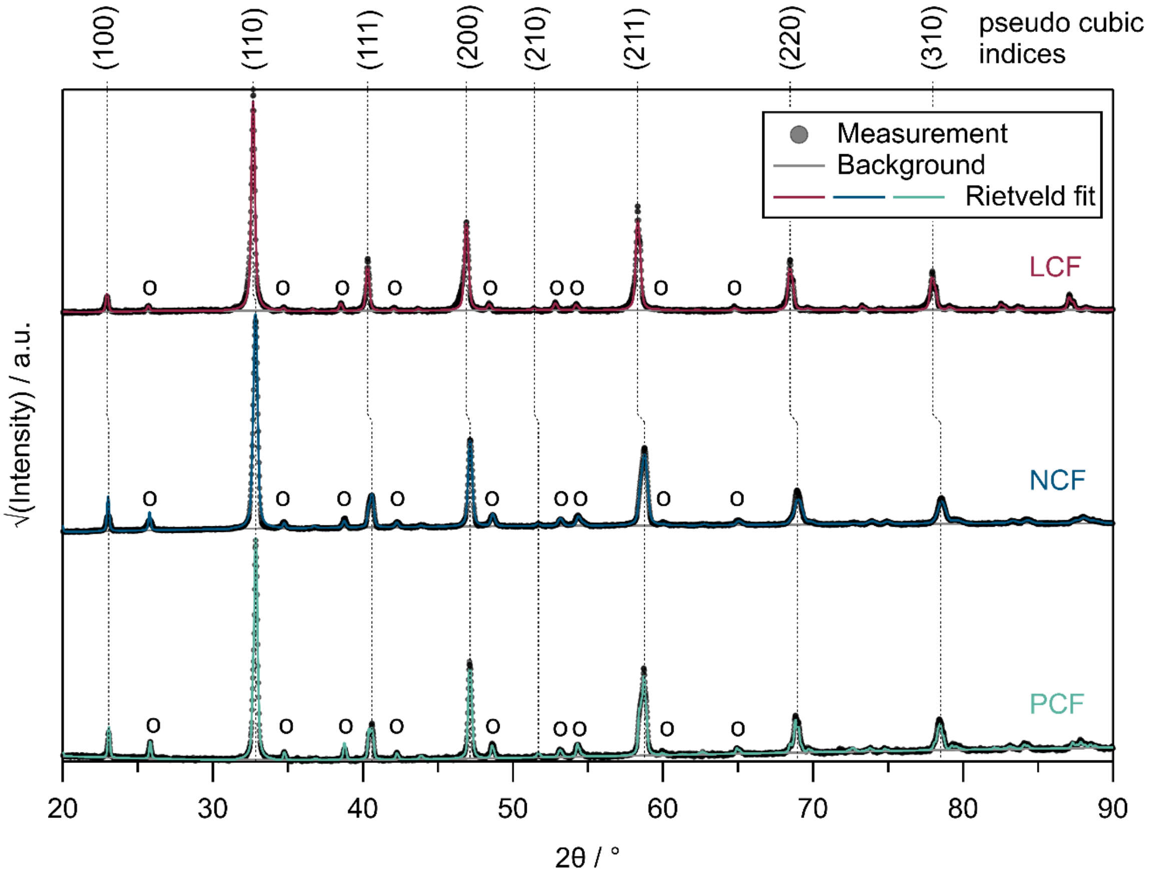

Figure S 1: Powder XRD measurements of LCF, NCF and PCF at ambient pressure and room temperature. All materials show phase purity. The displayed Miller indices are pseudo cubic. Reflexes markings labeled as ‘o’ indicate the additional reflexes caused by the orthorhombic structure.

Table S 1: Results from the Rietveld refinement. Note that the unit cells contain 4 Fe atoms. The value in parentheses represents the uncertainty in the last digit.

material	lattice parameters						unit cell volume / Å^3
	a / Å	b / Å	c / Å	α / °	β / °	γ / °	
LCF	5.478(7)	7.744(3)	5.478(7)	90	90	90	232.4(5)
NCF	5.480(5)	7.742(6)	5.479(9)	90	90	90	232.5(3)
PCF	5.477(3)	7.703(2)	5.436(8)	90	90	90	229.3(9)

RSF values for NAP-XPS quantification

Table S 2 shows the RSF values for the cation transitions used for quantification. The RSF_{pwdr} value for the Pr3d transition was not available. Hence, a value of 44.5 was assumed, calculated based on the average $RSF_{pwdr}/RSF_{Scofield}$ ratio of 0.8 for other measured lanthanides.

Table S 2: Relative Sensitivity Factors (RSF) for La3d, Nd3d, Pr3d, Ca2p and Fe2p transitions. For quantification, the RSF_{pwdr} values from Brundle et al.[31] were used. The RSF_{pwdr} value for the Pr3d transition, indicated by an asterisk (*), was unavailable and thus calculated based on the average $RSF_{pwdr}/RSF_{Scofield}$ ratios of other lanthanides.

transition	La3d	Nd3d	Pr3d	Ca2p	Fe2p
RSF_{pwdr}[31]	38.7	46.1	44.5*	6.15	14.5
$RSF_{Scofield}$[32]	47.6	59.3	55.6	5.07	16.4
$RSF_{pwdr}/RSF_{Scofield}$	0.81	0.78	0.80	1.21	0.88

Electrochemical Impedance Spectroscopy (EIS)

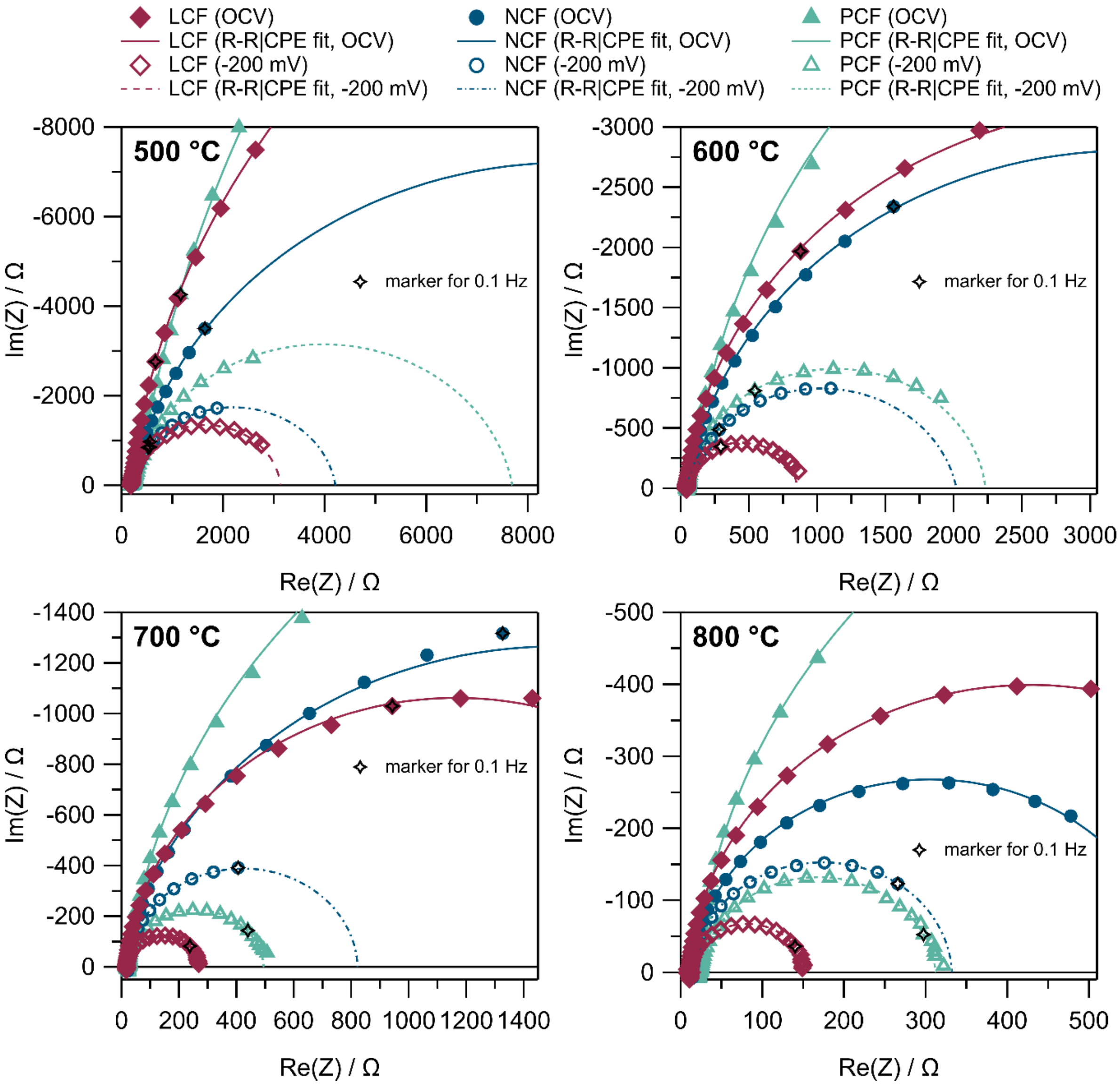

Figure S 2: Nyquist plots for LCF, NCF and PCF between 500 °C and 800 °C at OCV and −200 mV

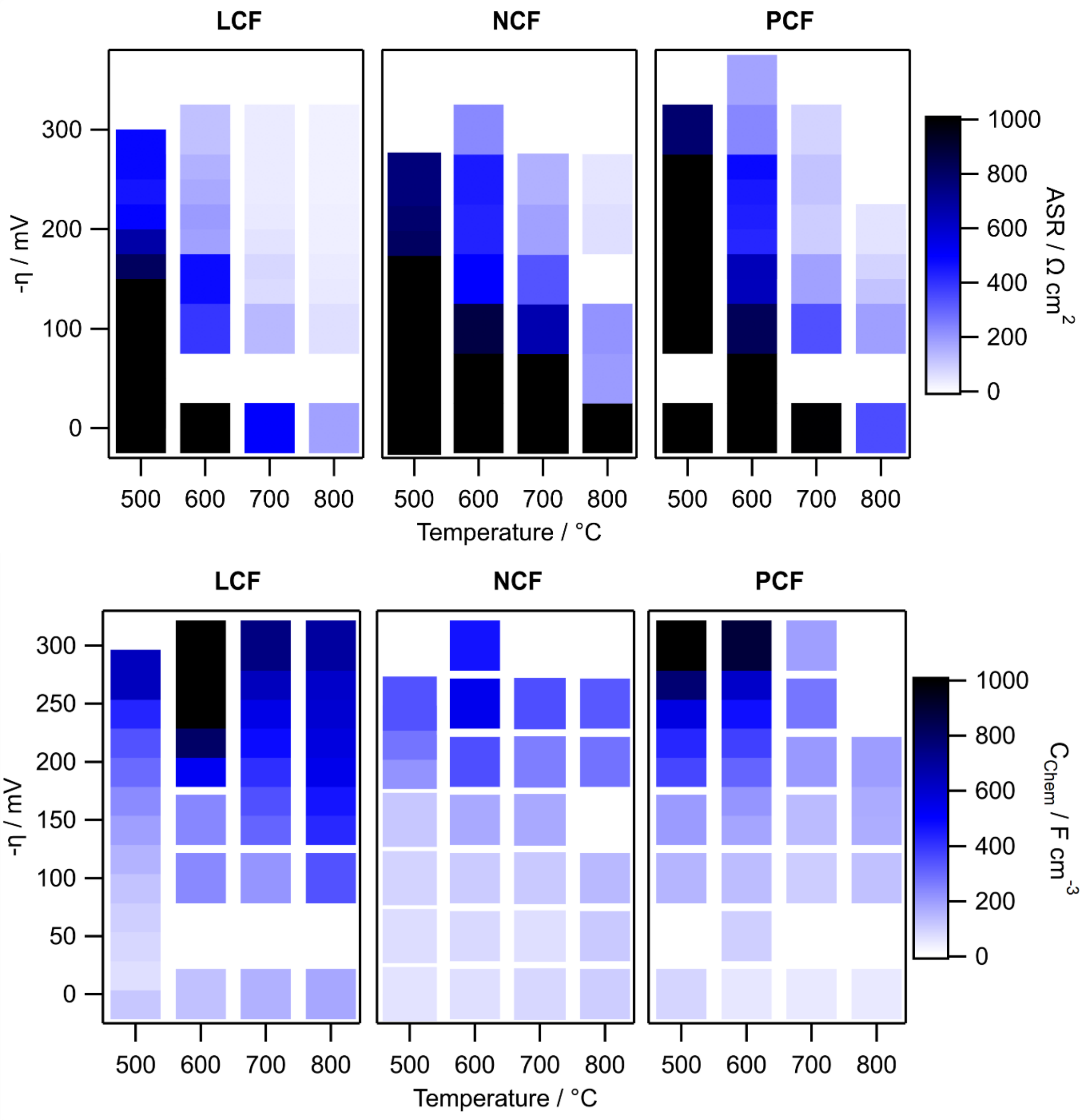

Figure S 3: Heat map for ASR and C_{chem} for LCF, NCF and PCF in dependence of temperature and overpotential

Hysteresis in U-I curves and Fe^0 fraction

The deviations from the hysteresis behavior of the current density of PCF at 800 °C and the Fe^0 amount of NCF at 800 °C in Figure S 4 shall be explained.

For PCF at 800 °C, no hysteresis is observed in the current density. This was somewhat purposely achieved by ramping the overpotential up to only −200 mV to confirm that when the amount of Fe^0 on the surface is very low, this hysteresis is not observed.

For NCF at 800 °C, a slight hysteresis in the current density is observed between −250 and −225 mV. In this range no Fe2p spectrum was recorded, therefore the hysteresis in the Fe^0 amount is not resolved because not enough data points had been collected.

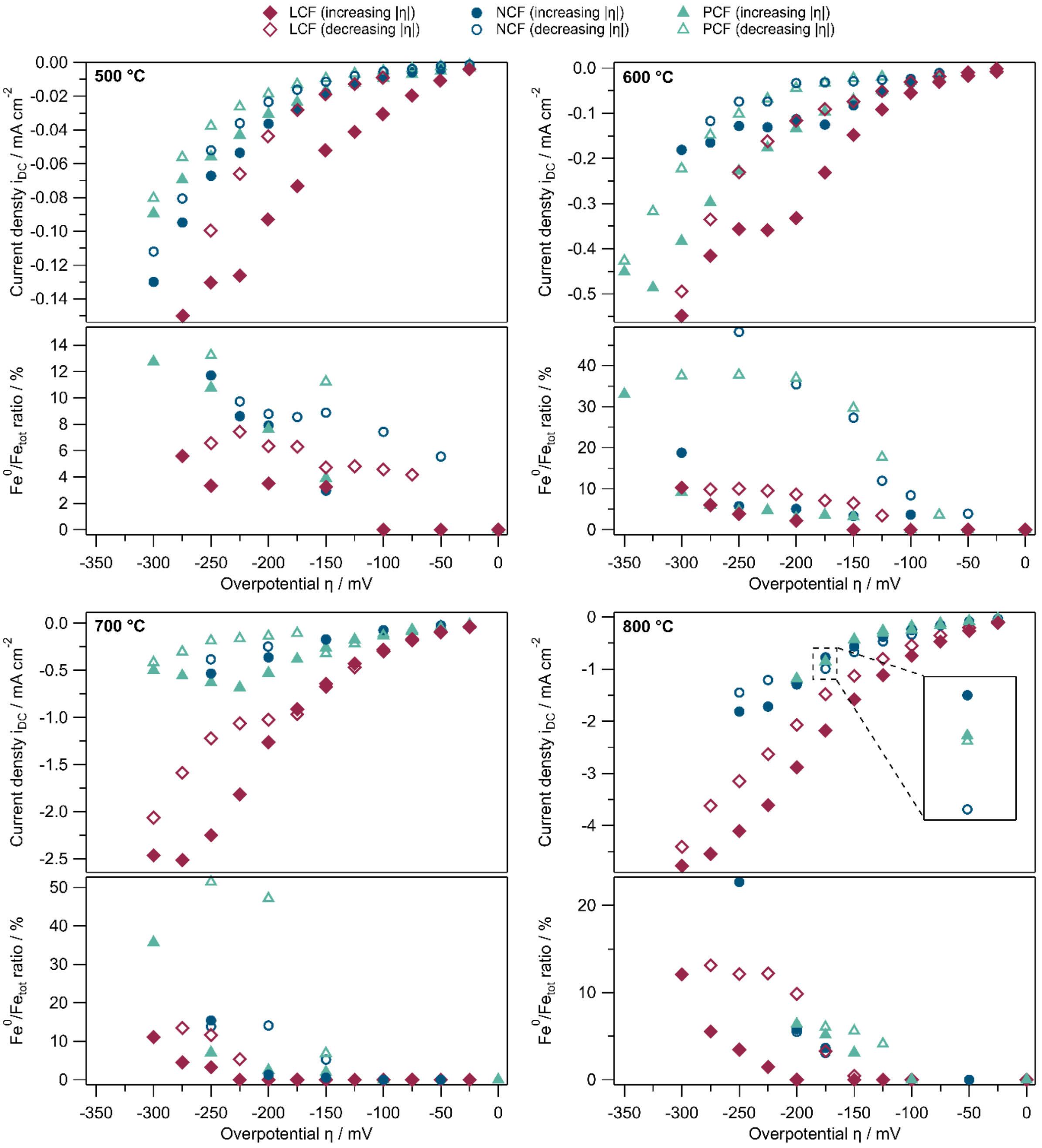

Figure S 4: Current-voltage characteristics (U-I curves) of LCF, NCF and PCF at various temperatures including the Fe^0/Fe_{tot} ratios for increasing and decreasing |η|

Amount of bulk exsolved Fe^0 for PCF at 700 °C

In Figure S5, looking at PCF at 700 °C, when the cathodic overpotential is decreased from −175 mV to −150 mV, a large current spike is observed in the time resolved I_{DC} measurements. The current even points in the opposite direction than the applied voltage. This behavior is explained by the large amount of exsolved Fe^0 observed in XPS measurements (~50 % Fe^0/Fe_{tot}). Therefore, when the overpotential is retraced back to more anodic values close to the Fe/FeO equilibrium, the metallic iron is re-oxidized electrochemically, leading to an anodic (positive) current. With numeric integration, the charge and therefore the number of exsolved Fe atoms can be calculated according to eq. (S1) and eq. (S2). The baseline (I_0) is linearly interpolated. The results are summarized in Table S1. Depending on the charge number (z), the calculation equates to 22.8 and 15.2 % for z = 2 and z = 3, respectively.

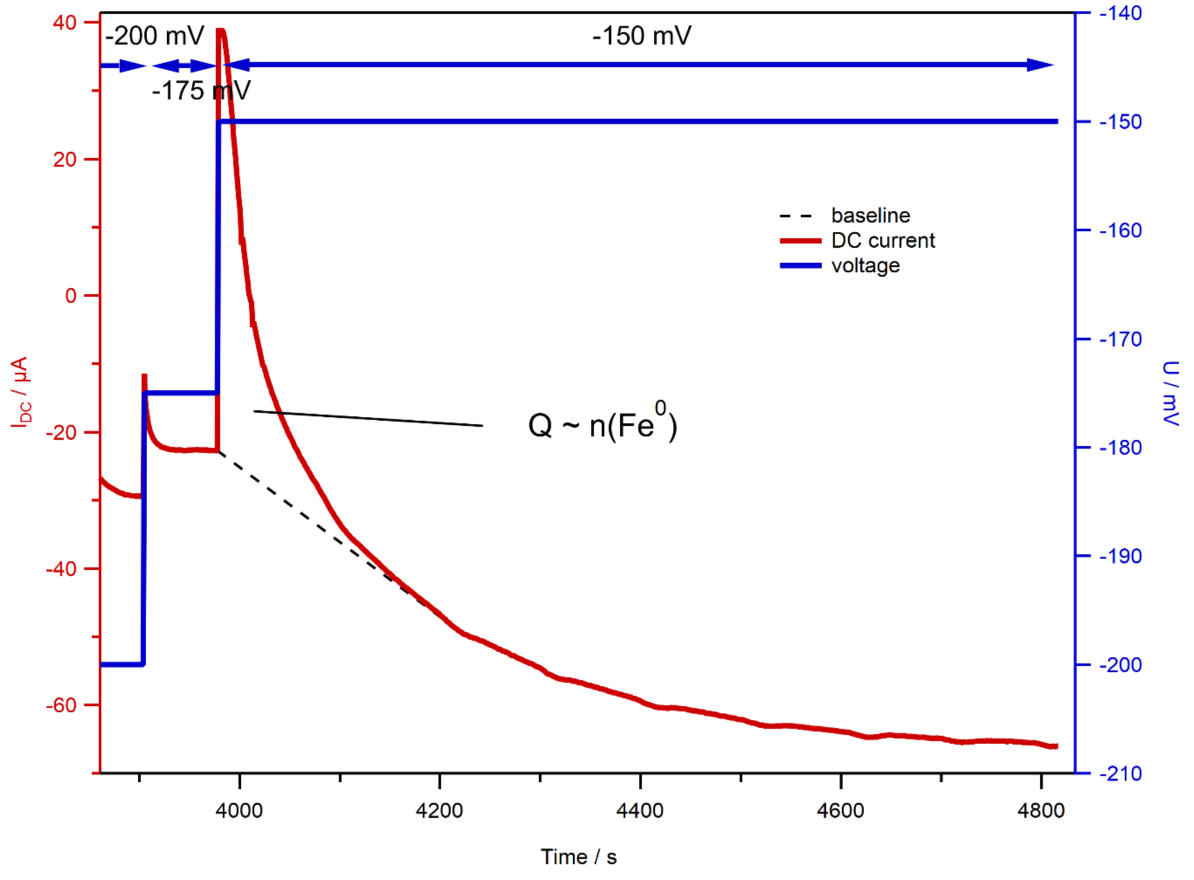

Figure S 5: Time-resolved DC current measurement for PCF at 700 °C with decreasing |η|. The integrated area is equal to the flown charge.

$$Q(t) = \int_{t0}^{t1} \left(I_{DC} - \bar{I}_0\right) dt \qquad \text{eq. (S1)}$$

$$n(Fe) = \frac{Q(t)}{zF} \qquad \text{eq. (S2)}$$

Table S 3: Amounts of re-oxidized Fe calculated from the DC current ($n(Fe)_{re\text{-}oxidized,\ DC\ current}$) for z = 2 and z = 3 with respect to the total amount of Fe in the PLD-layer calculated from XRD data ($n(Fe)_{tot,\ PLD\text{-}layer}$). Thickness of the PLD layer is 100 nm and the unit cell volume is 229.39 A^3.

reaction	charge number z	$n(Fe)_{tot,\ PLD\text{-}layer}$	$n(Fe)_{re\text{-}oxidized,\ current}$	ratio
$Fe + O^{2-} \rightleftharpoons FeO + 2e^-$	2	$5.7\cdot10^{-5}$ mmol	$1.3\cdot10^{-5}$ mmol	22.8 %
$Fe + \frac{3}{2}O^{2-} \rightleftharpoons \frac{1}{2}Fe_2O_3 + 3e^-$	3	$5.7\cdot10^{-5}$ mmol	$8.6\cdot10^{-6}$ mmol	15.2 %

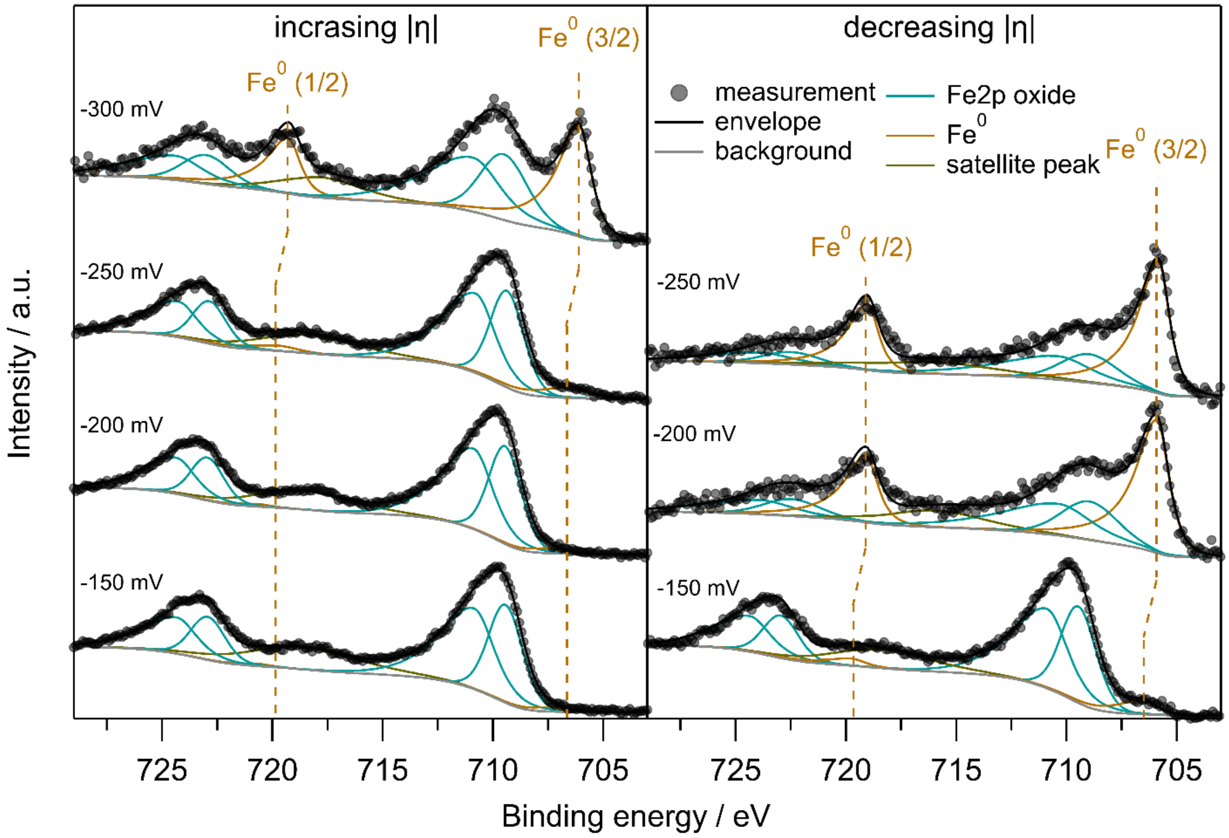

Figure S 6: Fe2p spectra of PCF at 700 °C with increasing (left) and decreasing |η| (right)

SEM Images and Cross Sections

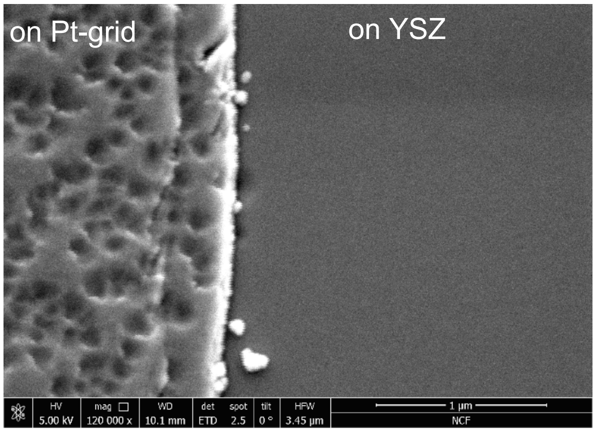

Figure S 7: SEM image of a pristine sample: PLD layer on top of Pt-grid to the left and on YSZ to the right.

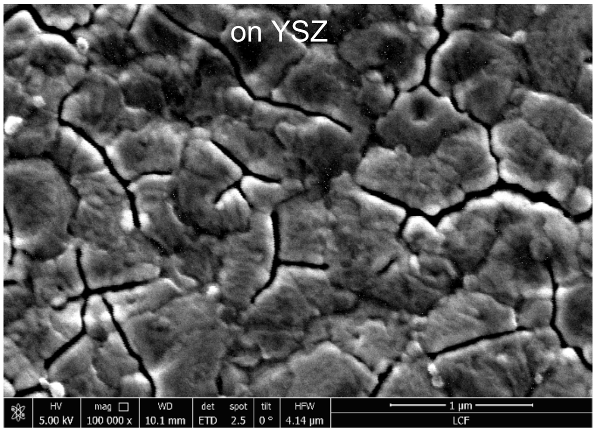

Figure S 8: SEM image of the PLD layer of LCF after the measurement at 700 °C in-between the Pt-grid (on YSZ).

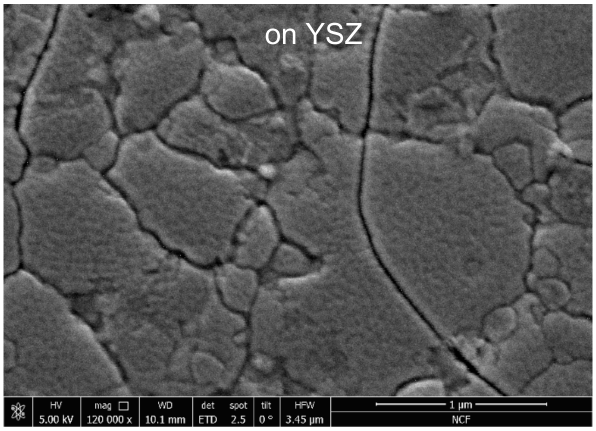

Figure S 9: SEM image of the PLD layer of NCF after the measurement at 700 °C in-between the Pt-grid (on YSZ).

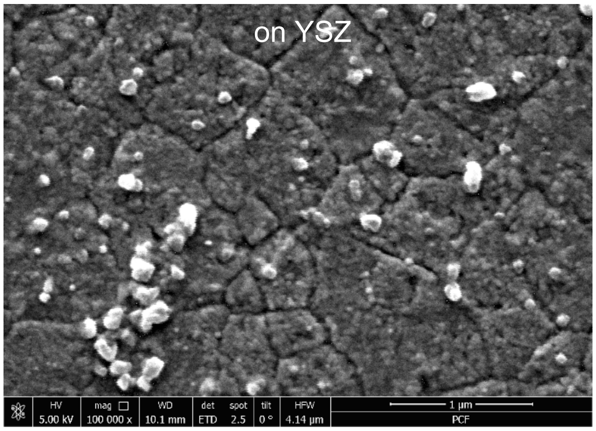

Figure S 10: SEM image of the PLD layer of PCF after the measurement at 700 °C in-between the Pt-grid (on YSZ).

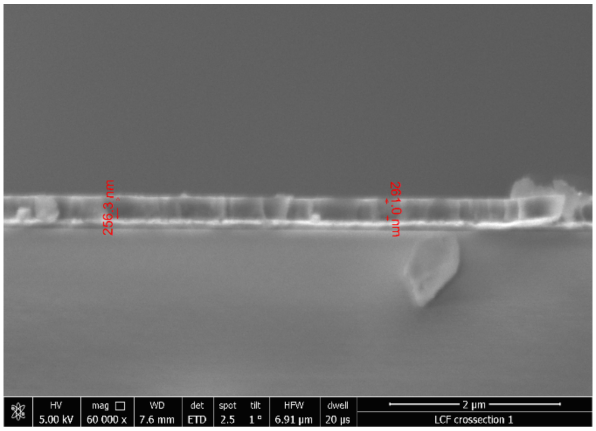

Figure S 11: Exemplary SEM cross section of LCF

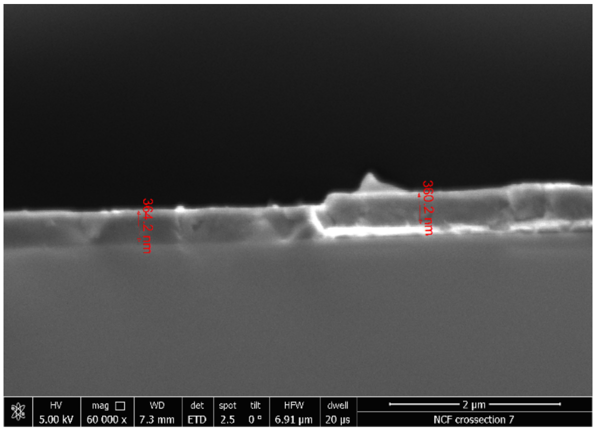

Figure S 12: Exemplary SEM cross section of NCF

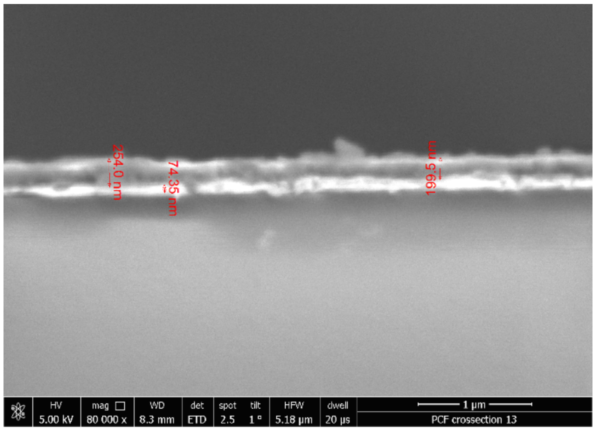

Figure S 13: Exemplary SEM cross section of NCF

Table S 4: Results of measured SEM cross sections of LCF, NCF and PCF thin-film model electrodes.

	thickness LCF / nm	thickness NCF / nm	thickness PCF / nm
average	245.9	368.8	254.4
standard deviation	13.6	23.0	25.8
